# Inducing a meditative state by artificial perturbations: A mechanistic understanding of brain dynamics underlying meditation

**DOI:** 10.1162/netn_a_00366

**Published:** 2024-07-01

**Authors:** Paulina Clara Dagnino, Javier A. Galadí, Estela Càmara, Gustavo Deco, Anira Escrichs

**Affiliations:** Computational Neuroscience Group, Center for Brain and Cognition, Department of Information and Communication Technologies, Universitat Pompeu Fabra, Barcelona, Spain; L’Hospitalet de Llobregat, Barcelona, Spain; Institució Catalana de la Recerca i Estudis Avancats (ICREA), Barcelona, Spain

**Keywords:** Meditation, fMRI, Brain states, Whole-brain modeling, Stimulation

## Abstract

Contemplative neuroscience has increasingly explored meditation using neuroimaging. However, the brain mechanisms underlying meditation remain elusive. Here, we implemented a mechanistic framework to explore the spatiotemporal dynamics of expert meditators during meditation and rest, and controls during rest. We first applied a model-free approach by defining a probabilistic metastable substate (PMS) space for each condition, consisting of different probabilities of occurrence from a repertoire of dynamic patterns. Moreover, we implemented a model-based approach by adjusting the PMS of each condition to a whole-brain model, which enabled us to explore *in silico* perturbations to transition from resting-state to meditation and vice versa. Consequently, we assessed the sensitivity of different brain areas regarding their perturbability and their mechanistic local-global effects. Overall, our work reveals distinct whole-brain dynamics in meditation compared to rest, and how transitions can be induced with localized artificial perturbations. It motivates future work regarding meditation as a practice in health and as a potential therapy for brain disorders.

## Author Summary

Our work explores brain dynamics in a group of expert meditators and controls. First, we characterized meditation and rest with a repertoire of brain patterns, each with its distinct probability of occurrence. Then, we generated whole-brain models of each condition, which enabled us to artificially perturb the systems to induce transitions between rest and meditation. Our results open new avenues in meditation research as a practice in health and disease.

## INTRODUCTION

*If we transform our way of perceiving things, then we transform the quality of our lives. This kind of transformation is brought about by the form of mind training known as meditation.*

Matthieu Ricard (Ricard & Singer, 2017)

Meditation encompasses a wide range of mental training techniques that allow one to explore the inner self and its relation with the outer world (Hanh, 1991; Kabat-Zinn, 2005). Meditation has roots in different cultures and religions, with its origin dating back to ancient Hindu and Buddhist spiritual traditions in India (Dumoulin, 1988; Sharma, 2015; Wynne, 2009). The different practices of meditation (e.g., attentional, open monitoring, loving kindness) have a variety of positive effects, including present-moment awareness and observation of experience with openness, acceptance, and nonattachment (Dahl, Lutz, & Davidson, 2015; Kabat-Zinn, 2003; Lutz, Slagter, Dunne, & Davidson, 2009; Ricard, Lutz, & Davidson, 2014). They can also promote stress reduction, well-being, social connectedness, self-awareness, and attention and emotional regulation (Basso, McHale, Ende, Oberlin, & Suzuki, 2019; Champion, Economides, & Chandler, 2018; Guendelman, Medeiros, & Rampes, 2017; Ludwig & Kabat-Zinn, 2008; Marchand, 2012; Pascoe, Thompson, Jenkins, & Ski, 2017; Tang, Hölzel, & Posner, 2015; Valentine & Sweet, 1999).

Over the past years, meditation is increasingly being explored as an intervention to enhance well-being and alleviate suffering in the healthy general population, as well as to prevent and recover from disability and disease (Dahl et al., 2015; Ludwig & Kabat-Zinn, 2008). The field of contemplative neuroscience, in particular, uses neuroscience tools—for example, functional magnetic resonance imaging (fMRI)—to study the effects of meditation (Davidson & Begley, 2013; Varela, 1993). Neuroanatomical studies have investigated structural gray matter thickness in meditators relative to controls, finding structural evidence such as the thickening of the prefrontal cortex with experience in long-term meditation practice (Lazar et al., 2005). Moreover, functional brain imaging studies have largely shown that key brain areas relevant to meditation belong to the default mode network (DMN), the salience network, and the control network (Fox et al., 2016; Ganesan et al., 2022; Hasenkamp, Wilson-Mendenhall, Duncan, & Barsalou, 2012; Sezer, Pizzagalli, & Sacchet, 2022). Interestingly, these resting-state networks belong to the triple-network model hypothesis which establishes their interaction as core to cognitive function (Menon, 2011). Moreover, this DMN-salience-control network interplay has been described in models of focused attention meditation developed by Hasenkamp et al. (2012) and Ganesan et al. (2022). Different static and seed-based approaches have analyzed blood oxygen level-dependent (BOLD) signal activity and network connectivity changes of meditation in health (Garrison, Scheinost, Constable, & Brewer, 2014; Ives-Deliperi, Howells, Stein, Meintjes, & Horn, 2013; Kilpatrick et al., 2011; Kyeong, Kim, Kim, Kim, & Kim, 2017; Martínez et al., 2021; Pagnoni, 2012; Vishnubhotla et al., 2021) and brain disorders (Datko et al., 2022; Goldin, Ramel, & Gross, 2009; Li et al., 2022; Lifshitz et al., 2019). Furthermore, theoretical methods have considered the underlying brain dynamics (Bremer et al., 2022; Lim, Teng, Patanaik, Tandi, & Massar, 2018; Martínez et al., 2021; Marusak et al., 2018; Mooneyham et al., 2017), including whole-brain approaches such as turbulent dynamics (Escrichs et al., 2022), dynamical complexity (Escrichs et al., 2019) and structural and effective connectivity (De Filippi et al., 2022). Whole-brain dynamic analysis and computational modeling can shed light on the mechanisms underlying meditation practices (Tang et al., 2015). Nonetheless, they remain largely unexplored.

The awakening framework developed by Deco et al. (2019) has been robust and helpful in elucidating the underlying mechanisms and brain transitions in different brain states towards control regimes (Deco et al., 2019; Escrichs et al., 2023; Mana et al., 2023; Vohryzek et al., 2024). It consists of a model-free approach, Leading Eigenvector Dynamics Analysis (LEiDA) (Cabral et al., 2017), and a model-based approach, consisting of Hopf whole-brain computational models and off-line *in silico* perturbations. Brain dynamics are studied with reference to the concept of metastability, which corresponds to the ability of a system to maintain equilibrium regardless of slight perturbations (Freyer, Roberts, Ritter, & Breakspear, 2012; Kelso, 2012). The LEiDA framework, in particular, typifies a brain state with its probabilistic metastable substate (PMS) space (Cabral et al., 2017; Deco et al., 2019; Escrichs et al., 2021; Figueroa et al., 2019; Kringelbach & Deco, 2020; Lord et al., 2019). Each PMS corresponds to a discrete repertoire of metastable substates (i.e., dynamic patterns) at critical points between chaos and order (Cabral et al., 2017; Deco, Kringelbach, Jirsa, & Ritter, 2017), in which substate duration and arrangement is a dynamic signature of a particular brain state (e.g., sleep, anesthesia) (Deco & Kringelbach, 2016; Tognoli & Kelso, 2014). By building a whole-brain model composed of a network of coupled local nodes (Botvinik-Nezer, Holzmeister, Camerer, et al., 2020; Deco et al., 2019), the empirical PMS can be simulated which then allows to force a transition to a desired control state with *in silico* external stimulation. Hence, whole-brain reactivity and its mechanistic underpinnings can be studied by stimulating one brain area at a time (Breakspear, 2017; Deco & Kringelbach, 2014; Deco, Tononi, Boly, & Kringelbach, 2015; Kringelbach & Deco, 2020).

Using the framework outlined above, we investigated the underlying mechanisms of brain activity during meditation. As a first step, we applied LEiDA to define the PMS of meditation and rest conditions. As a second step, we developed a Hopf whole-brain model based on the empirical PMS of each condition at the bifurcation point, where oscillatory and noisy regimes are undifferentiated, and the system is critical. Lastly, we applied off-line *in silico* external, unilateral, and localized probing to force transitions between conditions. Thus, we could evaluate local brain areas in terms of their sensitivity to stimulation and their mechanistic global effects after perturbation. Overall, our work contributes to the state of the art of contemplative neuroscience, as we try to understand brain mechanisms during meditation. Furthermore, it motivates current and potential future therapies based on meditation (Grossman, Niemann, Schmidt, & Walach, 2004; Ludwig & Kabat-Zinn, 2008). This way, we could benefit actual and future practices for healthy population and brain disorders.

## MATERIALS AND METHODS

### Participants

This study selected 20 experienced meditators and 20 healthy controls from a dataset previously described in Escrichs et al. (2019). The meditator group (i.e., expert meditators) consisted of 13 males and 7 females (mean age = 39.8 years (*SD* = 10.29); education = 13.6 years; and meditation experience = 9,526.9 hours (*SD* = 8,619.8). Meditators were recruited from Vipassana communities in Barcelona, Catalonia, Spain, and had more than 1,000 hours of meditation experience with a daily practice of over one hour. The healthy control group (i.e., controls) consisted of 13 males and 7 females with no prior meditation experience (mean age = 39.75 years (*SD* = 10.13); education = 13.8 years). Both groups were well-matched for age, gender, and educational level and reported no history of neurological disorder. All participants provided written informed consent, and the study was approved by the Ethics Committee of the Bellvitge University Hospital in accordance with the Helsinki Declaration.

### MRI Data Acquisition

Magnetic resonance imaging (MRI) scans were performed on a 3 T (Siemens TRIO) scanner using a 32-channel receiver coil. High-resolution T1-weighted images were acquired with 208 contiguous sagittal slices, with the following parameters: repetition time (TR) 1,970 ms, echo time (TE) 2.34 ms, inversion time (IT) 1,050 ms, flip angle 9°, field of view (FOV) 256 mm, and isotropic voxel size 1 mm. Resting-state and meditation functional MRI (fMRI) scans were obtained using a single-shot gradient-echo echo-planar imaging (EPI) sequence, comprising a total of 450 volumes, with TR 2,000 ms, TE 29 ms, FOV 240 mm, in-plane resolution 3 mm, 32 transversal slices, thickness 4 mm, and a flip angle of 80°. Diffusion-weighted imaging (DWI) data were collected using a dual spin-echo diffusion tensor imaging (DTI) sequence, comprising 60 contiguous axial slices, with TE 92 ms, FOV 236 mm, isotropic voxel size 2 mm, no gap, and matrix sizes 118 × 118. Diffusion was measured with 64 optimal noncollinear diffusion directions using a single b value of 1,500 s/mm^2^ interleaved with nine nondiffusion b0 images, with a frequency-selective fat saturation pulse applied to avoid artifacts.

### Experimental Conditions

In this study, we evaluated expert meditators scanned during meditation and rest, and controls scanned during rest. During rest, participants were instructed not to think about anything in particular. During meditation, participants had to focus on their natural breathing and return whenever their mind wandered (i.e., Anapanasati in Pali language). Participants were asked to remain still and move as little as possible inside the scanner. In the final contrast analysis, we excluded the first 90 volumes of the meditation fMRI condition to ensure that the initial acquisition stage corresponding to the acclimatization to the scanner, which may have interacted with the ability to enter a stable meditative state, did not influence the physiological measures collected. No consensus is established in meditation research with respect to the amount of time needed for entering a stable meditative state, and the decision was based on past studies considering enough remainer recording needed for a high-quality analysis (Braboszcz, Cahn, Levy, Fernandez, & Delorme, 2017; Davanger, Ellingsen, Holen, & Hugdahl, 2010; Manna et al., 2010). For normalization purposes, we applied the same criteria for each resting-state fMRI condition to have equal time points.

### Resting-State fMRI Preprocessing

Preprocessing was done using MELODIC version 3.14 (Beckmann & Smith, 2004), which is part of FMRIB’s Software Library (FSL, https://fsl.fmrib.ox.ac.uk/fsl). First, the initial 10 volumes were discarded for magnetization stabilization, followed by motion correction using MCFLIRT (Jenkinson, Bannister, Brady, & Smith, 2002), nonbrain removal using BET (Brain Extraction Tool) (Smith, 2002), spatial smoothing with 5-mm FWHM Gaussian Kernel, rigid-body registration, high-pass filter cutoff = 100.0 s, and single-session ICA with automatic dimensionality estimation. Noise removal was performed using FIX (FMRIB’s ICA-based X-noiseifier) (Griffanti et al., 2014), which independently removes noise components for each participant. FSLeyes in Melodic mode was used to manually classify the single-subject Independent Components (ICs) into “good” for signal, “bad” for noise, and “unknown” for ambiguous components, based on the spatial map, the time series, and the temporal power spectrum (Griffanti et al., 2017; Salimi-Khorshidi et al., 2014). Then, FIX was applied using the default parameters to obtain a cleaned version of the functional data. All participants showed low motion, given the mean framewise displacement (FD) was <2 mm (Power, Barnes, Snyder, Schlaggar, & Petersen, 2012). The mean and standard deviation for controls during rest, meditators during rest and meditators during meditation was 0.0149 ± 0.0052, 0.0113 ± 0.0023, and 0.0151 ± 0.0046, respectively.

We then used FSL tools to extract the time series in native EPI space between 100 cortical (Schaefer et al., 2018) and 16 subcortical brain nodes (Tian, Margulies, Breakspear, & Zalesky, 2020). Specifically, we co-registered the cleaned functional data to the T1-weighted structural image using FLIRT (Jenkinson & Smith, 2001). The T1-weighted image was then co-registered to the standard MNI space using FLIRT (12 DOF) and FNIRT (Andersson, Jenkinson, Smith, et al., 2007; Jenkinson & Smith, 2001). The resulting transformations were concatenated, inversed, and applied to warp the resting-state atlas from MNI space to the cleaned functional data in native space, preserving the labels with a nearest-neighbor interpolation method. Finally, we used custom-made MATLAB scripts to extract the averaged time series using fslmaths and fslmeants.

### Probabilistic Tractography Preprocessing

For each participant, a whole-brain structural connectivity matrix (SC) was computed using the atlas described above following the steps of previous studies (Cao et al., 2013; Gong et al., 2009; Muthuraman et al., 2016). The analysis was conducted using FMRIB’s Diffusion Toolbox (FDT) in FMRIB’s Software Library (FSL). First, the DICOM images were converted into Neuroimaging Informatics Technology Initiative (NIfTI) format using dcm2nii. The b0 image in native space was then co-registered to the T1-weighted image using FLIRT (Jenkinson & Smith, 2001). The co-registered T1 image was then co-registered to the standard space using FLIRT and FNIRT (Andersson et al., 2007; Jenkinson & Smith, 2001). The resulting transformation was inverted and applied to warp the atlas from the MNI space to the native MRI diffusion space using nearest neighbor interpolation. Next, the diffusion-weighted images were processed using the FDT pipeline in FSL. Nonbrain tissues were removed using the Brain Extraction Tool (BET) (Smith, 2002). The eddy current distortions and head motion were corrected using the eddy correct tool (Andersson & Sotiropoulos, 2016), and the gradient matrix was reoriented to correct for motion (Leemans & Jones, 2009). To model crossing fibers, BEDPOSTX was used, and the probability of multifibre orientations was computed to improve the sensitivity of nondominant fibre populations (Behrens, Berg, Jbabdi, Rushworth, & Woolrich, 2007; Behrens et al., 2003). Probabilistic Tractography was then performed for each participant in their native MRI diffusion space using the default settings of PROBTRACKX (Behrens et al., 2003, 2007). Finally, the connectivity probability *SC*_*np*_ between nodes *n* and *p* were calculated as the proportion of sampled fibres in all voxels in node *n* that reach any voxel in node *p*. Since diffusion tensor imaging (DTI) does not capture fiber directionality, the *SC*_*np*_ matrix was symmetrized by computing its transpose matrix *SC*_*pn*_ and averaging both matrices.

### Leading Eigenvector Dynamics Analysis (LEiDA)

**Figure 1.**
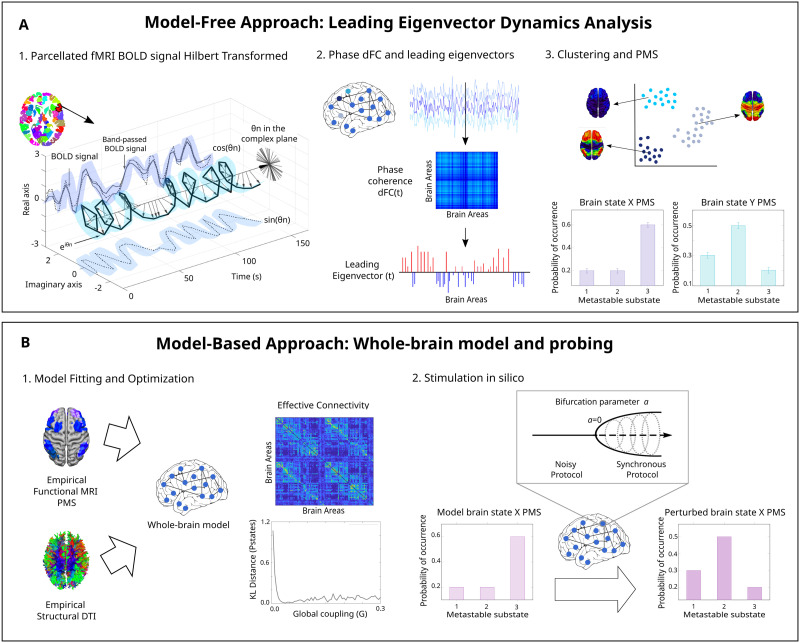
Methodology for model-free and model-based approaches. (A) Model-free framework: Leading Eigenvector Dynamic Analysis (LEiDA). In each parcellated brain area, the fMRI BOLD signal was band-passed filtered and Hilbert transformed to obtain the amplitude and phase information. For all participants in meditation and resting-state, the phase coherence matrix dFC(*t*) between brain areas was computed at each repetition time. The dimensionality of each matrix was reduced to its leading eigenvector *V*_1_(*t*) into positive (red color) and negative (blue color) values. Afterwards, K-means clustering was applied to the leading eigenvectors and the PMS computed for both meditation and resting-state. The optimal number of cluster centers was chosen according to the minimum value of *k* with the highest proportion of probabilities of occurrence of the metastable substates with significant difference between meditation and resting-state (schematized for *k* = 3). (B) Model-based framework: whole-brain model. A Hopf whole-brain computational model was built for resting-state using the empirical frequency *w* calculated on the empirical fMRI signal, and fitting the modeled PMS space to the empirical PMS by looking at the global coupling *G* that minimized their KL distance. Effective connectivity was used to optimize the model by adjusting the DTI with a gradient descent approach until convergence. *In silico* stimulation was applied in each brain node separately by moving the bifurcation parameter *a* positively for a synchronization protocol, and negatively for a noise protocol. Optimal transition was detected for the bifurcation parameter that gave the minimum KL distance between the perturbed modeled PMS, and the empirical PMS. Figure adapted from Deco et al. (2019) under Creative Commons Attribution License 4.0 (CC BY).

The first step was to define the empirical conditions from a quantitative point of view, by using the LEiDA method (Cabral et al., 2017). As outlined in Figure 1A, in each parcellated brain area the blood oxygenation level-dependent (BOLD) time series were filtered (0.04–0.07 Hz) and Hilbert-transformed. For all participants in resting-state and meditation, at each repetition time (TR), a BOLD phase coherence matrix—dynamical functional connectivity dFC(*t*)—was calculated between each brain area pair *n* and *p*. This was done by calculating the cosine of the phase difference:

$$dFC\left(n,p,t\right)=\cos\left(\theta \left(n,t\right)-\theta \left(p,t\right)\right).$$ (1)

When a pair of nodes are temporarily aligned, the phase coherence is close to one since the difference between their Hilbert transformed signal angle is 0° [cos(0°) = 1]. Contrarily, orthogonal BOLD signals have a phase coherence close to zero [cos(90°) = 0]. The resulting dFC(*t*) of each participant represented the interregional BOLD synchrony at each timepoint. The size of the matrix was N × N × T, where N is the number of brain areas (116) and T the total time points (350). A total of 14,000 (20 participants * 350 timepoints * 2 conditions) matrices (dimension N × N) were calculated. A dominant connectivity pattern was obtained by reducing the dimensionality of the undirected and symmetric matrices into their leading eigenvectors *V*_1_(t) (dimension N × 1) (Deco et al., 2019). The eigenvectors capture the dominant connectivity pattern at each time point *t*, representing the contribution of each brain area to the whole structure (Cabral et al., 2017). It is possible to reproduce the dominant connectivity pattern of dFC(*t*) by calculating the outer product of *V*_1_(t) and its transpose (V1.V1.^*T*^) (Lohmann et al., 2010). The leading eigenvectors of the dFC(*t*) for each TR and all participants from all states were then clustered with K-means clustering, varying *k* from 2 to 8. The resulting cloud centroids *V*_*c*_(t), which represent the dominant connectivity pattern in each cluster, were rendered onto a brain map using the Surf Ice software (https://www.nitrc.org/projects/surfice/).

Lastly, we computed the Probabilistic Metastable Substate Space (PMS) for each condition (i.e., rest and meditation). The PMS includes all the metastable substates extracted in LEiDA. Thus, each condition is characterized by a unique PMS corresponding to the probability of being in each metastable substate. Specifically, the ratio of number of TRs in a cluster divided by the total number of TRs in all clusters was computed separately for resting-state and meditation.

### Whole-Brain Computational Model

A whole-brain computational model was built for meditation and resting-state. These were generated using the normal form of a supercritical Hopf bifurcation. This Landau-Stuart oscillator, referred to as Hopf, has been used to study transitions from noisy to oscillatory regimes (Deco et al., 2015) (Figure 1B). Emergent brain dynamics were simulated by linking the structural and functional connectivity using effective connectivity (Deco et al., 2015). For each of the 100 cortical and 16 subcortical brain areas (Schaefer et al., 2018; Tian et al., 2020), BOLD activity was emulated and the working point was fitted and optimized with specific parameters of the model (Deco et al., 2019).

A normal form of a supercritical Hopf bifurcation (Landau-Stuart oscillator) was used to simulate the signal for each brain area. In complex coordinates, each node *n* was described by the following nonlinear equation:

$$\frac{\text{d}z_{n}}{\text{d}t}=z_{n}\left(a_{n}+i\omega_{n}-{\left|z_{n}\right|}^{2}\right)+\eta_{n},$$ (2)

where *η*_*n*_(*t*) is the additive Gaussian noise of *n*, *f*_*j*_ = *ω*_*n*_/2*π* is the frequency, and *a*_*n*_ is the local bifurcation parameter. This last parameter governs the dynamics of each brain node by changing qualitatively the nature of the solutions of the system. The Stuart-Landau equation can produce radically different signals depending on the value of *a*, for which a supercritical bifurcation is found at *a* = 0. When the bifurcation parameter is larger than zero (i.e., *a* > 0) there is a stable limit cycle oscillation of frequency *w*/2*π* and the system presents self-sustained oscillations. On the other hand, when the bifurcation parameter is much less than zero (i.e., *a* < 0), the local dynamics decay to a stable point (i.e., low activity noisy state) (Deco, Cabral, et al., 2017). In other words, a noise signal is generated, resulting from Gaussian noise added to a fixed point. A value just below zero, *a*_*n*_ = −0.02, was selected for each brain node *n* as it generates a fluctuating stochastically structured signal. This has been found to preserve resting-state network structure better and dynamically responding brain networks (Sanz Perl et al., 2023).

By replacing *z* with the complex numbers (*z*_*n*_ = *x*_*n*_ + *iy*_*n*_), the BOLD activity for a brain area *n* can be simulated with the following coupled equations in Cartesian coordinates (Deco, Cabral, et al., 2017):

$$\begin{array}{l} \frac{\text{d}x_{n}}{\text{d}t}=\left[a_{n}-x_{n}^{2}-y_{n}^{2}\right]x_{n}-\omega_{n}y_{n}+{\beta \eta }_{n}\left(t\right),\\ \frac{\text{d}y_{n}}{\text{d}t}=\left[a_{n}-x_{n}^{2}-y_{n}^{2}\right]y_{n}+\omega_{n}x_{n}+{\beta \eta }_{n}\left(t\right), \end{array}$$ (3)

where *x*_*n*_ and *y*_*n*_ correspond to the real and imaginary parts, respectively. In addition, *η*_*n*_(*t*) corresponds to the additive Gaussian noise, *β* = 0.01 to the standard deviation of the additive Gaussian noise, and *f*_*n*_ = *ω*_*n*_/2*π* is the frequency of the system. This frequency was estimated by averaging the empirically filtered BOLD signals in the 0.04- to 0.07-Hz band for each brain node *n* = 1, …, 116 (Deco et al., 2019).

Furthermore, a global coupling weight *G* and an additive coupling term *C*_*np*_ were included. The whole-brain dynamics at each node *n* were defined as:

$$\begin{array}{l} \frac{\text{d}x_{n}}{\text{d}t}=\left[a_{n}-x_{n}^{2}-y_{n}^{2}\right]x_{n}-\omega_{n}y_{n}+G\sum_{p_{=1}}^{N}C_{np}\left(x_{p}-x_{n}\right)+{\beta \eta }_{n}\left(t\right),\\ \frac{\text{d}y_{n}}{\text{d}t}=\left[a_{n}-x_{n}^{2}-y_{n}^{2}\right]y_{n}+\omega_{n}x_{n}+G\sum_{p_{=1}}^{N}C_{np}\left(y_{p}-y_{n}\right)+{\beta \eta }_{n}\left(t\right), \end{array}$$ (4)

where the global coupling weight *G* represents the strength between all nodes (i.e., axonal conductivity) and scales all of the connections equally. The additive coupling term *C*_*np*_ is built upon the SC and represents the input to node *n* from each of the rest of the nodes *p* (i.e., myelination density). It is a weighted matrix since it assumes different densities for each node.

#### Model fitting: Comparing empirical and simulated probability metastable space states.

In each whole-brain model, the global coupling weight *G* was ranged from 0 to 0.3 and the model run 200 iterations for each value of *G*. The simulated PMS space was then computed based on the centroids of the empirical clusters, which define the metastable substates. This ensured that the probabilistic measures reflected the metastable substates precisely. The best fit was obtained for the value of *G* that resulted in a maximal similarity between the modeled PMS at resting-state and the empirical PMS at resting-state, corresponding to their lowest Kullback-Leibler (KL) distance (Deco et al., 2019). This metric is given by:

$$KL\left(P_{emp},P_{sim}\right)=0.5\left(\sum_{i}P_{emp}\left(i\right)\ln\left(\frac{P_{emp}\left(i\right)}{P_{sim}\left(i\right)}\right)+\sum_{i}P_{sim}\left(i\right)\ln\left(\frac{P_{sim}\left(i\right)}{P_{emp}\left(i\right)}\right)\right),$$ (5)

where for each brain metastable substate *i*, *P*_*emp*_(*i*) are the empirical probabilities and *P*_*sim*_(*i*) the simulated probabilities.

#### Model optimization: Method for updating effective connectivity.

For each value of *G*, the SC was updated to address potential connections that were missing. The initial value of *C* corresponded to the average of the empirical DTI structural connectivity of all participants from the group being modeled (i.e., expert meditators or controls) normalized to 0.2 (Deco et al., 2019; Deco, Kringelbach, et al., 2017; Deco et al., 2015). The distance between the grand average phase coherence matrices of the model ${FC}_{ij}^{\text{phases}\_\text{mod}}$ and the empirical matrices ${FC}_{ij}^{\text{phases}\_\text{emp}}$ was calculated iteratively and the SC was transformed to effective connectivity (EC). A gradient descent approach adjusted each structural connection between each pair of nodes *i* and *j*:

$$C_{ij}=C_{ij}+\epsilon \left({FC}_{ij}^{\text{phases}\_\text{emp}}-{FC}_{ij}^{\text{phases}\_\text{mod}}\right),$$ (6)

in which *ϵ* = 0.01, and the grand average phase coherence matrix is given by:

$${FC}_{ij}=\langle \cos\left(\varphi_{j}\left(t\right)-\varphi_{i}\left(t\right)\right)\rangle .$$ (7)

The Hilbert transform BOLD signal phase of nodes *j* and *i* at time *t* is expressed by *φ*(*t*) and the brackets indicate the average across time. Each time, the empirical and simulated values were compared until the difference was less than 0.001 (Deco et al., 2019).

### Unilateral Perturbation of the Whole-Brain Model

Transitions between each source state and a target state (i.e., from resting-state toward meditation and vice versa) were evaluated as schematized in Figure 1B. The Hopf model allows shifting the bifurcation parameter of a particular brain area while keeping the rest of the regions at their bifurcation value corresponding to the optimal fit of the model (i.e., *a* = −0.02), thus representing a local perturbation. In other words, the whole-brain model for a source state was perturbed unilaterally by moving the local bifurcation parameter *a* of one brain area individually and separately while maintaining the rest fixed to their initial value. As a consequence of a local stimulation, the global brain dynamics of the perturbed model changed and were analyzed by calculating the perturbed PMS using the empirical cluster centroids found during LEiDA analysis. A measure of the fit to the target state was evaluated by computing the KL distance between the perturbed PMS and the target empirical PMS. The perturbation was repeated independently for each of the 116 brain areas. The areas with the lowest KL distance were the optimal to promote transitions. In order to minimize random effects from the Gaussian noise of the model, each simulation was repeated three times (Deco et al., 2019). There were two different protocols studied: synchronization and noise. For the synchronization protocol, the bifurcation parameter *a* of each brain area was shifted from −0.02 to 0.18 in steps of 0.01, given that for positive values of *a* there is a stable limit cycle oscillation. As for the noise protocol, the bifurcation parameter *a* of each brain area was shifted negatively from −0.02 to −0.22 in steps of 0.01 for the local dynamics to have a low activity noisy state. Stimulation intensities are represented by the absolute value of each step (Deco, Kringelbach, et al., 2017).

### Statistical Analysis

The statistical analysis was performed using MATLAB R2022a from MathWorks (Natick, MA, USA). To test the results of the PMS obtained by LEiDA, Wilcoxon tests with 1,000 iterations were used to examine the probability of occurrence of all explored clustering conditions (*k* from 2 to 8). Permutations were compared using the Wilcoxon test with a significance threshold of 0.05. Multiple comparisons were corrected with the False Discovery Rate (FDR) method (Hochberg & Benjamini, 1990) when testing the differences between resting-state and meditation for all metastable substates in each value of *k*. The reported *p* values remain significant after FDR correction.

## RESULTS

### LEiDA

#### Expert meditators during resting-state and meditation.

**Figure 2.**
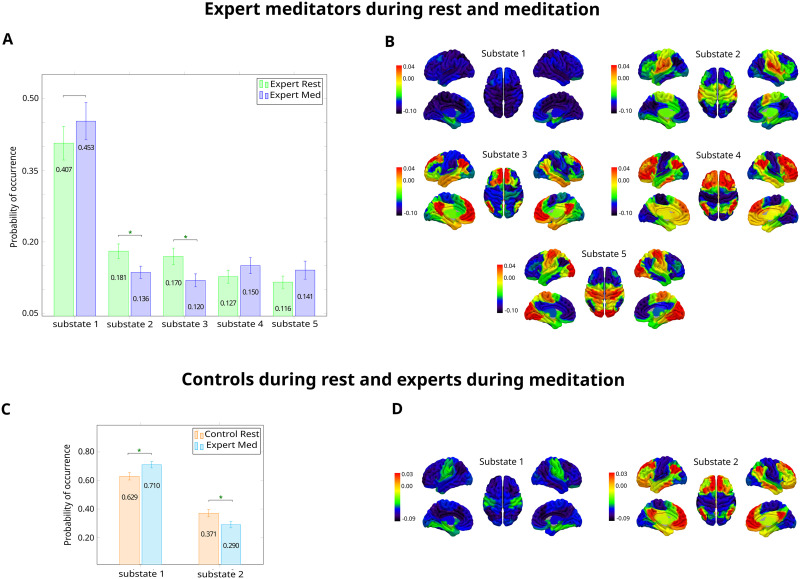
Leading Eigenvector Dynamic Analysis (LEiDA). (A) Empirical Probabilistic Metastable Substate (PMS) Space of expert meditators during resting-state and meditation. Mean probability of occurrence of resting-state and meditation in each metastable substate. Differences were computed with a 95% confidence interval and significance is represented with asterisks (*p < 0.05, **p < 0.01, and ***p < 0.001). Differences surviving multiple comparison correction are represented in green. Substates 1, 4, and 5 had a higher probability of occurrence during meditation compared to resting-state. Substates 2 and 3 presented the opposite behavior. (B) Expert meditators during resting-state and meditation. Cluster centroids *V*_*c*_(t) rendered onto brain maps, representing leading eigenvectors. Substate 1 had all eigenvector elements with the same sign. Substate 2 was characterized with an interaction mostly between the somatomotor network and salience network. Substate 3 presented a functional network dominated by the DMN and areas from the limbic system. Substate 4 had community formed mainly by the control network and the DMN. Substate 5 showed positive values mostly in areas from the visual system, and also the somatomotor network. (C) Empirical Probabilistic Metastable Substate (PMS) Space of controls during resting-state and expert meditators during meditation. Substate 1 had a higher probability of occurrence during meditation compared to resting-state. Substate 2 presented the opposite behavior. (D) Controls during resting-state and expert meditators during meditation. Leading eigenvectors of the two cluster centroids *V*_*c*_(t) rendered onto brain maps. Substate 1 had all negative eigenvector elements and Substate 2 had a community led by areas mainly from the DMN, and some from the control network and limbic system.

We first analyzed the group of expert meditators during resting-state and meditation. We selected the minimum number of *k* that provided the maximum significant differences between the probabilities from each condition for each given substate (i.e., *k* = 5). This selection survived correction by multiple comparisons and was the maximum value acceptable for the model-based analysis in terms of computational costs. We illustrate the probability of occurrence of the PMS of resting-state and meditation in Figure 2A. In addition, we rendered cluster centroid eigenvectors onto the brain maps where the sign of eigenvector elements are positive or negative and indicated by red and blue colors, respectively (Figure 2B). The signs represent the direction that the BOLD signals from different elements follow in relation to their projection onto the leading eigenvector. Hence, classifying elements into communities in which color strength reflects how strongly each area is associated with its community (Alonso Martinez, Deco, Ter Horst, & Cabral, 2020; Cabral et al., 2017). In the first metastable substate, all eigenvector elements had the same sign (negative). On the other hand, substate 2 showed a functional network dominated mainly by somatomotor and salience networks. Additionally, substate 3 displayed a community comprised mostly of the DMN and a few areas from the limbic network, whereas substate 4 showed coordination primarily between the control network and the DMN. Lastly, substate 5 had a network principally led by the visual system and some areas of the somatomotor network. Significant differences between conditions were found in the probability of occurrence of substates 2 and 3. For substate 2, the probability of occurrence was lower during meditation compared to resting-state [0.1364 ± 0.0129 vs. 0.1807 ± 0.0150, *p* = 0.0157]. The same applied for substate 3, given it was visited less often during meditation compared to rest [0.1196 ± 0.0136 vs. 0.1696 ± 0.0175, *p* = 0.0161]. Different clustering configurations can be consulted in Supporting Information Figure S1 and Figure S2. As a further analysis, we investigated the relationship between the probability of occurrence of each substate and the hours of experience of expert meditators. We calculated this for both rest and meditation, in separate analysis, in order to look whether the experience of experts meditators affected the probability of any substate during any condition. We found no significant correlations (Supporting Information Figure S3). Lastly, we looked for the correlation between the condition delta FD (meditation - rest) and the delta substate occupancy for each substate finding no significant results (Supporting Information Figure S4). This confirmed that there was no relationship between the head motion during the scan and the probability of occurrence of the substate led by the somatomotor network. Overall, LEiDA allowed us to elucidate distinct patterns of brain dynamics providing a more complete disentangling of brain configuration compared to classical static analysis (Supporting Information Figure S5).

#### Controls during resting-state and expert meditators during meditation.

We then analyzed differences between controls during rest and expert meditators during meditation. We found that the minimum number of *k* that provided a maximal proportion of statistically significant differences between conditions was *k* = 2 (Figure 2C). The centroid eigenvectors for this clustering configuration are rendered into brain maps in Figure 2D. The metastable substate 1 had all negative eigenvector elements, whereas substate 2 had a coordination led by brain areas from the DMN, and some from the control network and limbic network. All substates revealed significant differences between conditions. In the case of substate 1, the probability of occurrence during meditation was higher compared to rest [0.7099 ± 0.0221 vs. 0.6291 ± 0.0258, *p* = 0.0246]. Conversely, substate 2 was visited less often during meditation compared to rest [0.2901 ± 0.0221 vs. 0.3709 ± 0.0258, *p* = 0.0327]. Different clustering configurations can be consulted in Supporting Information Figure S6. Lastly, we sought to determine if there is a relationship between the probability of occurrence of each substate during meditation and the hours of practice of expert meditators. No significant correlation was found (Supporting Information Figure S3). Furthermore, the correlation between the condition delta FD (meditation - rest) and the delta substate occupancy for each substate did not have any significant result (Supporting Information Figure S4). Head motion was not associated with substate occupancy.

### Whole-Brain Computational Models

#### Expert meditators during resting-state and meditation.

**Figure 3.**
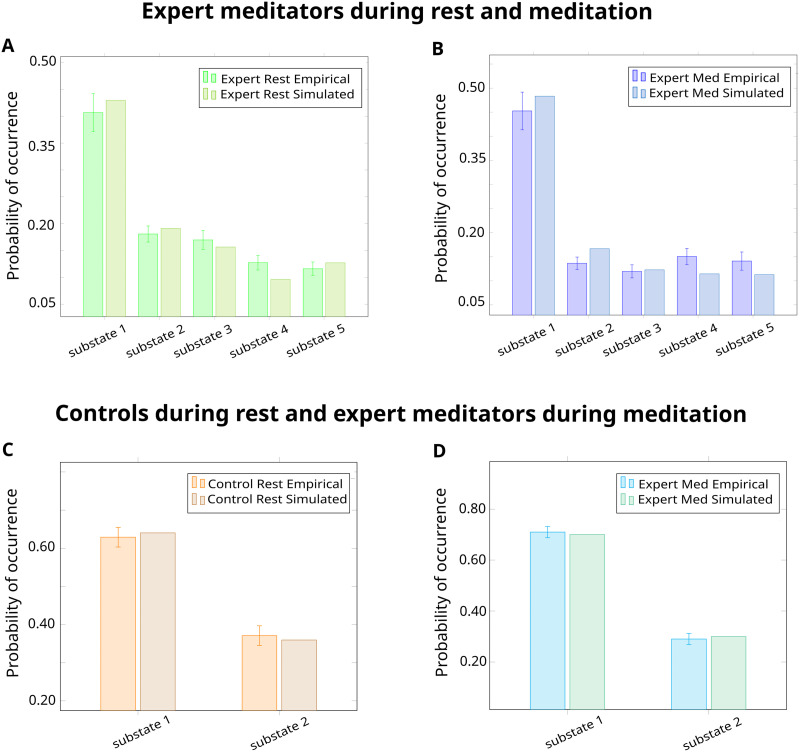
Model-based results: Whole-brain model. Empirical and simulated PMS in the analysis of expert meditators during resting-state and meditation: (A) for resting-state, *G* = 0.04 with a KL distance of 0.0064; (B) for meditation, *G* = 0.05 with a KL distance of 0.0122. Empirical and simulated PMS in the analysis of controls during resting-state and meditation: (C) for resting-state, *G* = 0.04 with a KL distance of 0.0003; (D) for meditation, *G* = 0.04 with a KL distance of 0.0002.

We fitted and optimized a whole-brain model for each condition in order to find the parameters that maximally approximated the corresponding empirical PMS. This was identified with the minimal KL distance between the simulated and the empirical PMS. The best fit for the models of meditation and resting-state were found at a global coupling of *G* = 0.05 with a KL distance of 0.0122, and *G* = 0.04 with a KL distance of 0.0064, respectively. The similar probabilities of occurrence for all five substates are shown in Figure 3A and Figure 3B. The modeled PMS for *k* = 4 can be consulted in Supporting Information Figure S7. The evolution of *G* as a function of the KL distance for each model can be consulted in Supporting Information Figure S8.

#### Controls during resting-state and expert meditators during meditation.

The best fit for the models of meditation and resting-state was found at a global coupling of *G* = 0.04 with a KL distance of 0.0002, and *G* = 0.04 with a KL distance of 0.0003, respectively. The simulated probabilities of the two substates are shown in Figure 3C and Figure 3D. The evolution of *G* as a function of the KL distance for each model can be consulted in Supporting Information Figure S8.

### *In Silico* Stimulations to Force Transitions

#### Expert meditators during resting-state and meditation.

In the last step of the analysis, we systematically perturbed the model of each source state and compared the resulting perturbed PMS with the empirical PMS of the target state. For the synchronous protocol, we shifted the bifurcation parameter *a* independently in each brain area, with positive values. Contrarily, for the noise protocol, we used negative values. We identified the optimal perturbation as the one resulting in the smallest KL distance between the perturbed modeled source PMS and the target empirical PMS.

**Figure 4.**
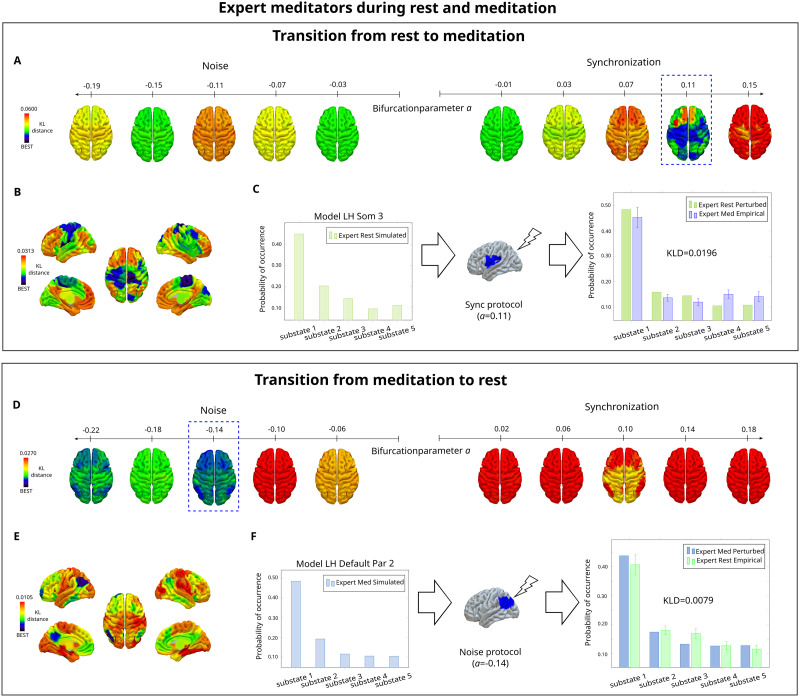
Model-based results: *In silico* stimulation. We stimulated one brain area at a time to study whole-brain effects and evaluate a possible transition between conditions. (A) The local bifurcation parameter *a* was shifted independently for each node using positive values in the synchronization protocol, and negative values in the noise protocol. Transitions from rest toward meditation were successful in the synchronization protocol. The *x*-axis represents the bifurcation and the color scale shows the KL distance between the perturbed PMS and the target PMS. In the noise protocol, the KL distance between the perturbed modeled PMS of resting-state and the empirical PMS of meditation, never decreased for increasing negative values of *a*. (B) Optimal perturbation for each brain area in the synchronization protocol, found at the stimulation intensity that gives the minimum KL between the modeled PMS of resting-state after perturbation, and the empirical PMS of meditation. Color code represents the KL distance. (C) Best fit was found when stimulating the area LH Som 3 from Schaefer 100 parcellation (Schaefer et al., 2018) with a bifurcation parameter value of *a* = 0.11. PMS for simulated resting-state, perturbed resting-state and empirical meditation. When the resting-state model was perturbed, the PMS of the perturbed rest state approximated the PMS of the empirical meditative state. (D) Contrarily, transitions from meditation to rest were successful in the noise protocol. (E) Optimal perturbation for each brain area in the noise protocol. (F) Best fit after stimulating the area LH Default Par 2 from Schaefer 100 parcellation (Schaefer et al., 2018) with a bifurcation parameter value of *a* = −0.14. When the meditation model was perturbed, the PMS of the perturbed meditative state approximated the PMS of the empirical rest state.

Results for perturbing the whole-brain model of resting-state in the synchronization and noise protocols at different stimulation intensities are illustrated in Figure 4A. The color-scale indicates the KL distances between the empirical and the perturbed PMS after stimulating each brain area separately. A transition was successful in the synchronization protocol, where KL distances decreased with increasing positive bifurcation point values. The minimum KL distance for the optimal perturbation of each brain area is rendered into the brain maps of Figure 4B. The top 10 areas to perturb, at their particular optimal stimulation intensity in the synchronization protocol, belonged mainly to the somatomotor network, posterior dorsal attentional network, and medial salience network in decreasing order of prevalence (Supporting Information Table S1). We achieved the best fit when perturbing the third segment from the left somatomotor cortex with *a* = 0.11. Consequently, the perturbed and target PMS were very similar, as reflected in Figure 4C.

On the other hand, transitions from meditation towards resting-state were successful in the noise protocol and not in the synchronization protocol. Here, KL distances decreased with decreasing negative values of the bifurcation parameter *a* (Figure 4D). The optimal stimulation for each brain area at a particular optimal stimulation intensity for the noise protocol can be seen in Figure 4E, in which the top 10 areas to perturb were found mainly in the DMN, followed by the control and limbic networks as well as the left anterior thalamus (Supporting Information Table S1). The optimal transition was found for the second segment of the left parietal region from the DMN for *a* = −0.14 (Figure 4F). Perturbational analysis for *k* = 4 can be consulted in Supporting Information Figure S9 and Supporting Information Table S2.

#### Controls during resting-state and expert meditators during meditation.

**Figure 5.**
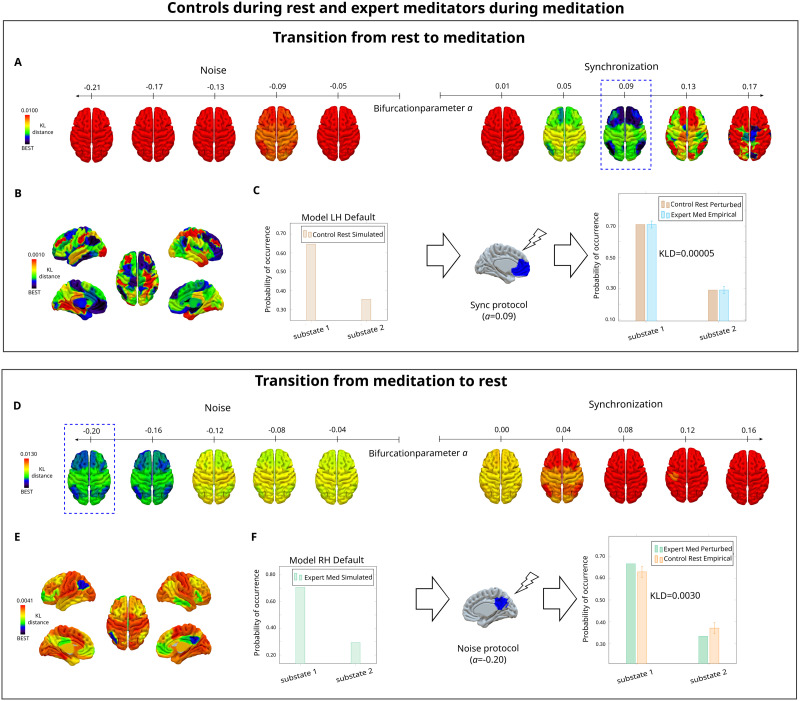
Model-based results: *In silico* stimulation in the analysis of controls during resting-state and meditation. (A) Successful transitions from rest toward meditation were obtained in the synchronization protocol and not in the noise protocol. This was determined by the lower and higher values of the KL distance between the perturbed modeled PMS of rest and the empirical PMS of meditation, respectively. (B) Minimum KL distances rendered onto brain maps for the synchronization protocol, given at the optimal stimulation intensity for each brain area. (C) Optimal transition obtained after perturbing the Default PFC three from Schaefer 100 parcellation (Schaefer et al., 2018) with a bifurcation parameter value of *a* = 0.09. The PMS of the perturbed resting-state is closer to the PMS of the empirical meditative state. (D) Transitions from meditation to rest were achieved in the noise protocol. (E) KL distances rendered onto brain maps for the optimal perturbation of each brain area in the noise protocol, at a particular simulation intensity. (F) Optimal transition at a bifurcation parameter value of *a* = −0.20 in the area RH Default PCunCC 2 from Schaefer 100 parcellation (Schaefer et al., 2018). The PMS of the perturbed meditative state is closer to the PMS of the target empirical rest state.

Transitions from rest to meditation were successful with the synchronization protocol Figure 5A. The optimal perturbation for each brain area in the synchronization protocol is shown in Figure 5B. Here, the top 10 areas to perturb were mainly from the DMN, and some brain areas from the somatomotor, limbic and control networks, and the subcortical areas left amygdala and left hippocampus (Supporting Information Table S3). The optimal transition was found when stimulating the third segment of the left prefrontal cortex from the DMN for *a* = 0.09 (Figure 5C).

Furthermore, results for stimulating the meditation model are illustrated in Figure 5D. Transitions were achieved in the noisy protocol and not in the synchronization protocol, with decreasing KL distance with more negative stimulation intensities. The best transition for each brain area is shown in Figure 5E, and the top 10 areas to perturb were mainly from the DMN, and the rest from the control network (Supporting Information Table S3). The optimal transition was found when perturbing the second segment of the right precuneus posterior cingulate cortex from the DMN for *a* = −0.20 (Figure 5F).

## DISCUSSION

We successfully studied brain dynamics using model-free and model-based approaches (Deco, Kringelbach, et al., 2017) to find mechanistic explanations for meditation. LEiDA allowed us to characterize brain signatures of meditation and resting-state by defining their PMS, with an associated probability of occurrence of being in distinct metastable substates (i.e., dynamic patterns) (Cabral et al., 2017). Then, we fitted a Hopf whole-brain computational model to the empirical PMS of each state. Consequently, this allowed us to force transitions from a modeled PMS to an empirical target PMS using *in silico* perturbations. By varying stimulation intensities and protocols (i.e., noise and synchronization), we were able to determine the mechanistic global effects of exhaustive local perturbations. This way, we revealed how local changes can alter whole-brain dynamics and evaluated the optimal transitions toward a given state. Using a synchronization protocol, we could transition from the resting-state to the meditative state. Furthermore, using a noisy protocol, we could transition from meditation to rest.

In the model-free approach, we used LEiDA to reveal signatures of functional networks underlying meditation and resting-state (Figure 2). In the analysis of expert meditators, we found a significantly lower probability of occurrence in substates 2 and 3 during meditation compared to rest. Firstly, we found that substate 2 was led by the somatomotor and salience networks. The somatomotor network is responsible for processing bodily sensations and motor functions (Biswal, Zerrin Yetkin, Haughton, & Hyde, 1995). According to the review of Ganesan et al. (2022), most published evidence on areas from the somatomotor network showed deactivation during meditation. We suggest that the decrease in this network could align with the disengagement from external sensory interferences in the practice. Furthermore, a reduction of somatomotor cortex activity has been associated with decreased pain-related perception in a meditative state (Nakata, Sakamoto, & Kakigi, 2014; Zeidan et al., 2011). Additionally, the salience network monitors the switching between internal and external processing (Elton & Gao, 2014; Goulden et al., 2014). Reduced functional connectivity within the salience network has been associated with experiential acceptance and present-moment awareness (Sezer et al., 2022). Guidotti et al. (2023) used a trained classifier revealing that this network was able to discriminate meditation styles in an expert group of meditators. On the other hand, substate 3 displayed a community led majorly by the DMN and some areas of the limbic network. The DMN is involved in internal thought, self-related processing, and mind wandering (Alves et al., 2019; Mason et al., 2007). Our result aligns with previous studies reporting a reduction in DMN activity during meditation (Ganesan, Moffat, Van Dam, Lorenzetti, & Zalesky, 2023; Garrison, Zeffiro, Scheinost, Constable, & Brewer, 2015). This reduction has been associated with the phenomenological well-being of meditation (Brandmeyer & Delorme, 2021; Killingsworth & Gilbert, 2010). Furthermore, relevant areas from the limbic network were the orbitofrontal cortex and the temporal pole, involved in emotional processing. The orbitofrontal cortex is engaged in reward-based tasks (Kringelbach & Rolls, 2004), whereas the temporal pole has a role in social processing (Herlin, Navarro, & Dupont, 2021). In turn, due to increased attention to the breath, we suggest the reduction in probability of this substate may decrease emotional and/or reward-related processes.

In the analysis of controls and expert meditators, we found a significantly higher probability of occurrence of substate 1 and a significantly lower probability of occurrence of substate 2 during meditation compared to rest. The first substate, characterized by all negative eigenvector elements, has been associated to synchronization, stability, or noise artifacts (Farinha, Amado, Morgado, & Cabral, 2022; Olsen et al., 2022). On the other hand, substate 2 resembled substate 3 from the previous analysis, and could be also explained by lower mind wandering (Mason et al., 2007) and emotional interference (Ganesan et al., 2022) during meditation.

For the model-based approach, we first built a Hopf whole-brain computational model for each condition. This allowed us to systematically evaluate *in silico* stimulations to force transitions between resting-state and meditation (Deco, Kringelbach, et al., 2017). In particular, we fitted each model to the empirical data and identified the optimal global coupling value, corresponding to the lowest KL distance between the empirical PMS and the modeled PMS (Figure 3). We then explored local perturbations area by area in order to study mechanistic global effects. We evaluated a synchronization protocol (i.e., toward a system with self-sustained oscillations) and a noise protocol (i.e., toward a system that decays to a stable point and low noisy activity). In the analysis of expert meditators, transitions from resting-state to meditation were successful with a synchronous protocol (Figure 4A–C). Our results show that the most sensitive areas to perturbation were found in the somatomotor network, posterior regions of the dorsal attention network, and medial areas from the salience network. Interestingly, these brain areas overlapped with substate 2 from the LEiDA analysis. In the somatomotor network, a sensitive area was the supplementary motor area, related to inner speech processing (Hertrich, Dietrich, & Ackermann, 2016). Its role here could be supported by the shift from internal distraction during meditation. Moreover, the dorsal attention network is associated with self-directed attention (Devaney et al., 2021). Its increased connectivity has been related to mindfulness and focused attention (Froeliger et al., 2012; Zhang et al., 2021). As a final point, the sensitivity of the salience network is highly relevant given it is hypothesized to play a modulator role in neurocognitive models of focused attention meditation (Ganesan et al., 2022; Hasenkamp et al., 2012). On the other hand, our results show that transitions from meditation to the resting-state were achieved in the noise protocol (Figure 4D–F). Here, the most sensitive areas belonged to the DMN, overlapping substate 3 in the LEiDA analysis. The role of the DMN in forcing a transition could be related to mind wandering and increased DMN activity at rest (Mason et al., 2007).

For the model-based results of controls and expert meditators, transitions from rest to meditation were also effective in the synchronization protocol (Figure 5A–C). Furthermore, highest sensitivity was found in the DMN and subcortical areas (i.e., hippocampus and amygdala). The sensitivity of the DMN for forcing a transition could be associated with its active role during rest, which decreases in meditative states (Mason et al., 2007). On the other hand, the hippocampus and amygdala have been associated with the emotional brain. The hippocampus is involved in emotional behavior, learning, memory, and spatial navigation (Anand & Dhikav, 2012). In addition, the amygdala plays a key role in the assignment of sensory information with emotional value (Šimić et al., 2021). Thus, their sensitivity in the transitions could be explained by the need to reduce interference from emotional information during meditation. On the other hand, our results show that transitions from meditation to the resting-state were achieved with a noisy protocol (Figure 5D–F). The most sensitive areas belonged to the DMN. The importance of the DMN has been revealed throughout the whole analysis, and the perturbational results show its pivotal influence on shifting transitions between rest and meditation. Lastly, the remaining most sensitive areas belonged to the central executive network. This network has a role in executive functions (i.e., attention, cognitive control, and working memory) (Niendam et al., 2012), and its influence on the transition could be related to the shift in cognitive demands from meditation to rest.

In all cases, a transition from rest to meditation was successful in a synchronization protocol, and the opposite transition occurred in a noise protocol. A fundamental brain function is short- and large-scale neural communication achieved with synchronization of oscillations between different brain areas (Schnitzler & Gross, 2005). Synchronization is essential to cognition, and abnormal values are associated with brain disorders (Uhlhaas & Singer, 2006). The relationship between synchronization and a meditative state is not straightforward. EEG literature of expert meditators during meditation showed increased gamma synchronization (Berkovich-Ohana, 2017; Lutz, Greischar, Rawlings, Ricard, & Davidson, 2004) and higher betweenness centrality in the alpha band (van Lutterveld et al., 2017). These could support our results given they were related to a restructuring in information processing and an increase of integrative mechanisms of information exchange for facilitating a meditative state (Engel, Fries, & Singer, 2001; Fell, Axmacher, & Haupt, 2010). In contrast, the transition from meditation to rest in the noisy protocol reflects that neural noise enables the emergence of the dynamic repertoire of the brain at rest (Ghosh, Rho, McIntosh, Kötter, & Jirsa, 2008), promoting less synchronization across the whole brain (Deco et al., 2019). Overall, our model-based results provide novel insights into how local perturbations influence global brain dynamics. We could explore different stimulation protocols and the most sensitive brain areas in terms of their response to perturbations as a model-based biomarker of the underlying mechanisms of meditation.

This study has some limitations. Firstly, we analyzed a relatively small sample size dataset. Therefore, validating our results using a larger dataset would be beneficial to increase the generalizability of our findings (Fox et al., 2016). We also want to acknowledge the fact that no established consensus on the time for the preparatory period in meditation research is yet established. The choice for the time removed due to the build-up period was drawn from past studies (Braboszcz et al., 2017; Davanger et al., 2010; Manna et al., 2010). In addition, our analysis focuses on a specific meditation technique that prevents the translation of our findings to all styles of meditation. Thus, we cannot oversimplify our results to one signature of brain dynamics in meditation given the heterogeneity of the practice (Fox & Cahn, 2019; Young et al., 2021). Moreover, complementary studies would be necessary to evaluate the longitudinal effects of meditation practice on brain structure and function.

In this work, we explored the underlying brain dynamics in meditation. Using the LEiDA model-free approach, we could quantitatively define the brain dynamics during meditation and resting-state. In addition, the model-based approach allowed us to measure the whole-brain reactivity to localized perturbation at resting-state and study transitions toward meditation and vice versa. As a last remark, we want to emphasize the importance of not oversimplifying the Eastern tradition of meditating to a single motivation around attention and emotion regulation (Garcia-Campayo, López Del Hoyo, & Navarro-Gil, 2021). And, if used as a therapeutic intervention, it is essential to recognize that meditation is not a cure-all solution (Wright, 2013). Overall, given the paucity of research in meditation with neuroscience tools, our results shed light on the field of contemplative neuroscience for promoting meditation practices in health and a potential therapy in disease.

## ACKNOWLEDGMENTS

We are sincerely grateful to Mind and Life Europe for supporting this project with the Francisco J. Varela Award. We thank participants for their involvement in the study.

## SUPPORTING INFORMATION

Supporting information for this article is available at https://doi.org/10.1162/netn_a_00366.

## AUTHOR CONTRIBUTIONS

Paulina Clara Dagnino: Conceptualization; Formal analysis; Investigation; Methodology; Project administration; Software; Validation; Visualization; Writing—original draft; Writing—review & editing. Javier A. Galadí: Writing—review & editing. Estela Càmara: Data curation; Resources; Writing—review & editing. Gustavo Deco: Conceptualization; Funding acquisition; Investigation; Methodology; Project administration; Resources; Software; Supervision; Validation; Writing—review & editing. Anira Escrichs: Conceptualization; Data curation; Formal analysis; Funding acquisition; Investigation; Methodology; Project administration; Resources; Software; Supervision; Validation; Writing—original draft; Writing—review & editing.

## FUNDING INFORMATION

P.D. was supported by the AGAUR FI-SDUR Grant (no. 2022 FISDU 00229) and by the AGAUR research support grant (ref. 2021 SGR 00917) funded by the Department of Research and Universities of the Generalitat of Catalunya. J.G. was supported by the Spanish Agencia Estatal de Investigación of the Ministerio de Ciencia e Innovación (FJC2021-047765-I). A.E. and G.D. were supported by the Grant PID2022-136216NB-I00 funded by MICIU/AEI/10.13039/501100011033 and by “ERDF A way of making Europe,” ERDF, EU. G.D. was also supported by the project NEurological MEchanismS of Injury, and Sleep-like cellular dynamics (NEMESIS) (ref. 101071900) funded by the EU ERC Synergy Horizon Europe. A.E. was also supported by the project eBRAIN-Health - Actionable Multilevel Health Data (id 101058516), funded by the EU Horizon Europe.

## Supplementary Material



## TECHNICAL TERMS

Functional magnetic resonance imaging (fMRI): Noninvasive neuroimaging technique for mapping brain activity in vivo based on relative changes in blood oxygen levels in the capillary beds.
Structural connectivity: Anatomical links between different brain areas, often measured with diffusion tensor imaging.
Effective connectivity: Directed influence of one brain area on another, not necessarily showing direct connections but measuring the interactions between different brain areas.
Brain state: Evolving pattern of self-organized neural activity specific to a condition.
Metastable substate: Dynamic large-scale functional network patterns that change over time, stochastic subdivisions of brain states.
Diffusion tensor imaging (DTI): Noninvasive magnetic resonance imaging technique that measures the brain’s white matter tracts in vivo based on the diffusion properties of water through nerve fibers.
Phase coherence matrix: Cosine difference of phases between each pair of brain areas.
Hopf bifurcation: In nonlinear dynamics, critical point where a system shifts from a fixed stable point toward a self-sustained oscillatory regime.
Functional connectivity: Statistical dependencies between different brain areas, often measured with the coherence and correlation between the time series from fMRI.
