# Supplementary material for: Inducing a meditative state by artificial perturbations: A mechanistic understanding of brain dynamics underlying meditation
Source: Netw Neurosci. 2024 Jul 1;8(2):517–40. doi: 10.1162/netn_a_00366 (PMC11168722; doi:10.1162/netn_a_00366)
Supplement: Supplementary file 1 [file netn-8-2-517-s001.pdf]

Journal: **NETWORK NEUROSCIENCE**

RESEARCH

Inducing a meditative state by artificial perturbations: A mechanistic understanding of brain dynamics underlying meditation

Paulina Clara Dagnino[1], Javier A. Galadí[1], Estela Càmara[2], Gustavo Deco[1,3#], and Anira Escrichs[1#]

[1]Computational Neuroscience Group, Center for Brain and Cognition, Department of Information and Communication Technologies, Universitat Pompeu Fabra, Barcelona, Catalonia, Spain

[2]Bellvitge Biomedical Research Institute (IDIBELL), Cognition and Brain Plasticity Unit

[3]Institució Catalana de la Recerca i Estudis Avancats (ICREA), Barcelona, Catalonia, Spain

[#]These authors share senior authorship

SUPPORTING INFORMATION

Expert meditators in resting-state and meditation (*k*=5)							
Resting-state to meditation				Meditation to resting-state			
Node label	x	y	z	Node label	x	y	z
LH SomMot 3	70	130	182	LH Default Par 2	205	62	83
RH SomMot 8	74	130	187	LH Default pCunPCC 2	205	63	85
LH SalVentAttn Med 2	197	58	252	LH Default PFC 4	205	63	80
RH SomMot 7	74	130	186	RH Cont Par 2	234	147	36
LH DorsAttn Post 6	0	118	19	LH Limbic TempPole 2	220	248	167
LH SomMot 5	70	130	184	LH aTHA	-8	-14	8
RH SalVentAttn Med 1	197	58	251	RH Default PFCv 1	209	61	78
LH DorsAttn Post 4	0	118	17	RH Cont PFCmp 1	234	148	38
RH DorsAttn Post 3	4	119	17	LH Default PFC 1	205	63	76
RH Vis 8	124	18	141	RH Default Par 1	209	62	78

Table. S 1. Top 10 most sensitive regions for transitions between brain states. Analysis of expert meditators in resting-state and meditation (*k*=5). Node labels and coordinates following Schaefer parcellation (Schaefer et al., 2018) for cortical areas and Tian parcellation (Tian et al., 2020) for subcortical areas. Node label defined as: hemisphere, resting-state network, [i] segment of the corresponding resting-state network. Abbreviations: LH – left hemisphere; RH – right hemisphere; Vis - Visual Network; SomMot - Somatomotor Network; DorsAttn - Dorsal Attention Network; SalVentAttn - Salience/Ventral Attention Network; Limbic - Limbic Network; Cont - Control Network; Default - Default Network; Post - posterior; Med - medial; TempPole - temporal pole; Par - parietal; PFC - prefrontal cortex; pCunPCC - precuneus posterior cingulate cortex; PFCv - ventral prefrontal cortex; aTHA - anterior thalamus.

Expert meditators in resting-state and meditation (*k*=4)							
Resting-state to meditation				Meditation to resting-state			
Node label	x	y	z	Node label	x	y	z
LH SomMot 3	70	130	182	LH Default pCunPCC 2	205	63	85
RH SomMot 4	74	130	183	RH Cont PFCl 2	233	148	35
LH DorsAttn Post 2	0	118	15	RH Default Par 1	209	62	78
RH SalVentAttn Med 1	200	57	250	RH Default pCunPCC 2	208	62	82
LH SomMot 5	70	130	184	RH Cont pCun 1	234	148	39
RH DorsAttn Post 2	4	119	16	LH Default Par 1	205	62	82
LH SomMot 1	70	130	179	RH Cont PFCl 4	233	148	37
RH SalVentAttn FrOperIns 1	201	59	252	RH DorsAttn PrCv 1	5	118	15
RH SomMot 1	74	130	179	LH Default Par 2	205	62	83
LH DorsAttn Post 4	0	118	17	LH Default PFC 2	205	63	77

Table. S 2. Top 10 most sensitive regions for transitions between brain states. Analysis of expert meditators in resting-state and meditation (*k*=4). Node labels and coordinates following Schaefer parcellation (Schaefer et al., 2018) for cortical areas and Tian parcellation (Tian et al., 2020) for subcortical areas. Node label defined as: hemisphere, resting-state network, [i] segment of the corresponding resting-state network. Abbreviations: LH – left hemisphere; RH – right hemisphere; Vis - Visual Network; SomMot - Somatomotor Network; DorsAttn - Dorsal Attention Network;Cont - Control Network; Default - Default Network; Post - posterior; PrCv - precentral ventral; FrOperIns - frontal operculum insula; PFCl - lateral prefrontal cortex; Med - medial; Par - parietal; pCun - precuneus; PFC - prefrontal cortex; pCunPCC - precuneus posterior cingulate cortex.

Expert meditators in resting-state and meditation (*k*=2)							
Resting-state to meditation				Meditation to resting-state			
Node label	x	y	z	Node label	x	y	z
LH Default PFC 3	205	63	79	RH Default pCunPCC 2	208	62	82
LH AMY	-22	-4	-18	LH Default Par 2	205	62	83
RH Default Par 1	209	62	78	RH Cont Cing 1	234	148	37
LH Default Par 2	205	62	83	LH Default PFC 4	205	63	80
LH Limbic TempPole 2	220	248	167	RH Default Temp 1	209	61	79
RH Cont PFCl 4	233	148	37	RH Default PFCv 2	209	61	82
LH HIP	-26	-22	-14	LH Cont Cing 1	231	149	36
RH SomMot 5	74	130	184	RH Default PFCdPFCm 1	208	62	79
RH Default PFCdPFCm 2	208	62	80	LH Cont pCun 1	231	149	35
LH SomMot 6	70	130	185	LH Default PFC 2	205	63	77

Table. S 3. Top 10 most sensitive regions for transitions between brain states. Analysis of controls in resting-state and expert meditators during meditation (*k*=2). Node labels and coordinates following Schaefer parcellation (Schaefer et al., 2018) for cortical areas and Tian parcellation (Tian et al., 2020) for subcortical areas. Node label defined as: hemisphere, resting-state network, [i] segment of the corresponding resting-state network. Abbreviations: LH – left hemisphere; RH – right hemisphere; Vis - Visual Network; SomMot - Somatomotor Network; SalVentAttn - Salience/Ventral Attention Network; Limbic - Limbic Network; Cont - Control Network; Default - Default Network; PFCl - lateral prefrontal cortex; TempPole - temporal pole; Par - parietal; pCun - precuneus; Cing - cingulate ; Temp - temporal ; PFC - prefrontal cortex; pCunPCC - precuneus posterior cingulate cortex; PFCv - ventral prefrontal cortex; PFCdPFCm - dorsal and medial prefrontal cortex; HIP - hippocampus; AMY - amygdala.

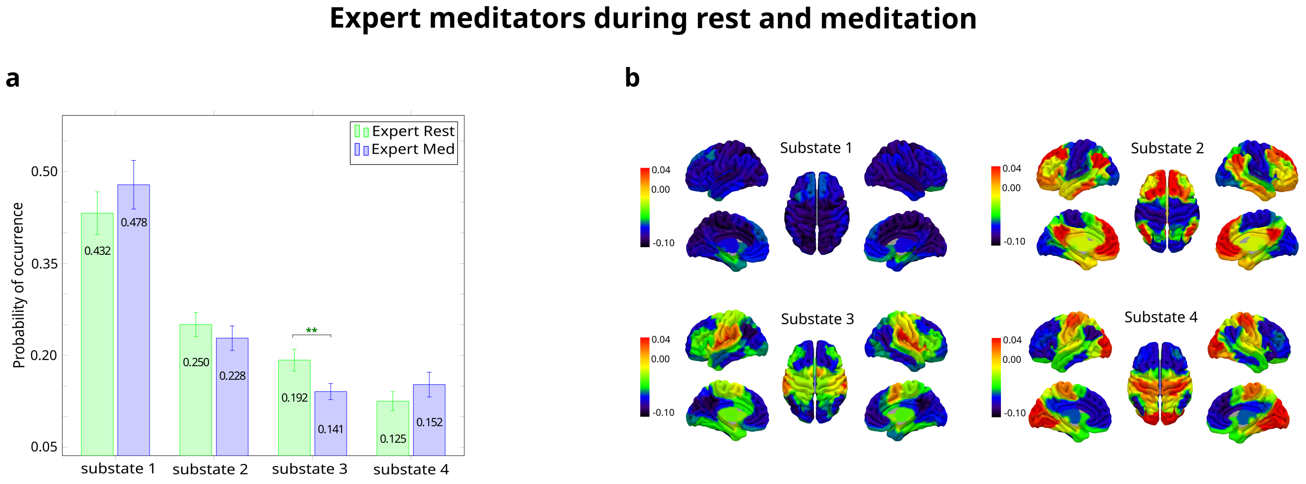

Figure. S 1. LEiDA for *k*=4 in the analysis of expert meditators during resting-state and meditation. **a** Empirical Probabilistic Metastable Substate (PMS) Space of resting-state and meditation. Differences were computed with a 95% confidence interval and significance is represented with asterisks (* p < 0.05, ** p < 0.01 and *** p < 0.001). Differences surviving multiple comparison correction are represented in green. Substates 1 and 4 had a higher probability of occurrence during meditation compared to resting-state. Substates 2 and 3 presented the opposite behavior. **b** Leading eigenvectors of the cluster centroids $V_c(t)$ rendered onto brain maps. The leading communities found for each substate similarly overlapped the ones revealed for *k*=5 of the main analysis, highlighting the robustness of the empirical LEiDA approach for different numbers of cluster centers. Furthermore, it reveals the clear characterization of resting-state and meditation brain dynamics in expert meditators. Metastable substate 1 was characterized by all negative eigenvector elements, as in substate 1 of *k*=5. In addition, substate 2 was led mainly by areas from the DMN, and very few from the control network and limbic system. Furthermore, substate 3 had a community dominated by the somatomotor and salience networks. Here, substate 2 and substate 3 could be related with substate 3 and substate 2 from *k*=5, respectively. This relation differs in terms of the overall probability of occurrence in each PMS: the substate led by the DMN had a higher probability of occurrence in *k*=4, whereas the substate led by the somatomotor and salience network has a higher probability of occurrence in *k*=5). The last substate 4 had a functional network led by the visual system and the somatomotor network, and could be closely related with substate 5 in *k*=5. The probability of occurrence of meditation compared to resting-state was significantly lower in substate 3 [0.1410 ± 0.0132 vs. 0.1924 ± 0.0177, *P*=0.0063].

Expert meditators during rest and meditation

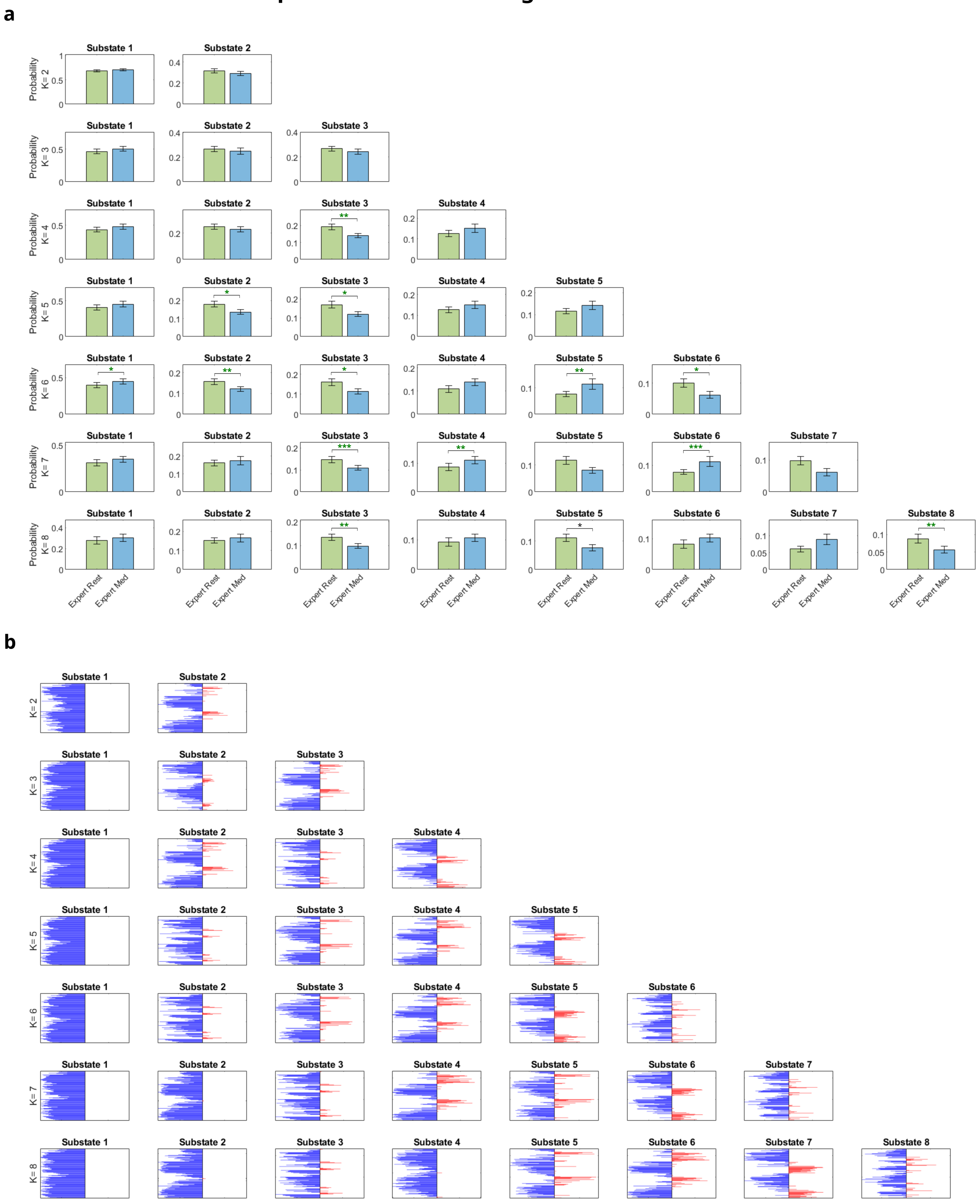

Figure. S 2.

Leading Eigenvector Dynamic Analysis (LEiDA) of expert meditators during resting-state and meditation for all values of *k*. **a** Empirical Probabilistic Metastable Substate (PMS) Space of resting-state and meditation. Differences were computed with a 95% confidence interval and significance is represented with asterisks (* $p < 0.05$, ** $p < 0.01$ and *** $p < 0.001$). Differences surviving multiple comparison correction are represented in green, while those not surviving correction are represented in black. **b** Leading Eigenvectors for each substate of the PMS for all values of *k*.

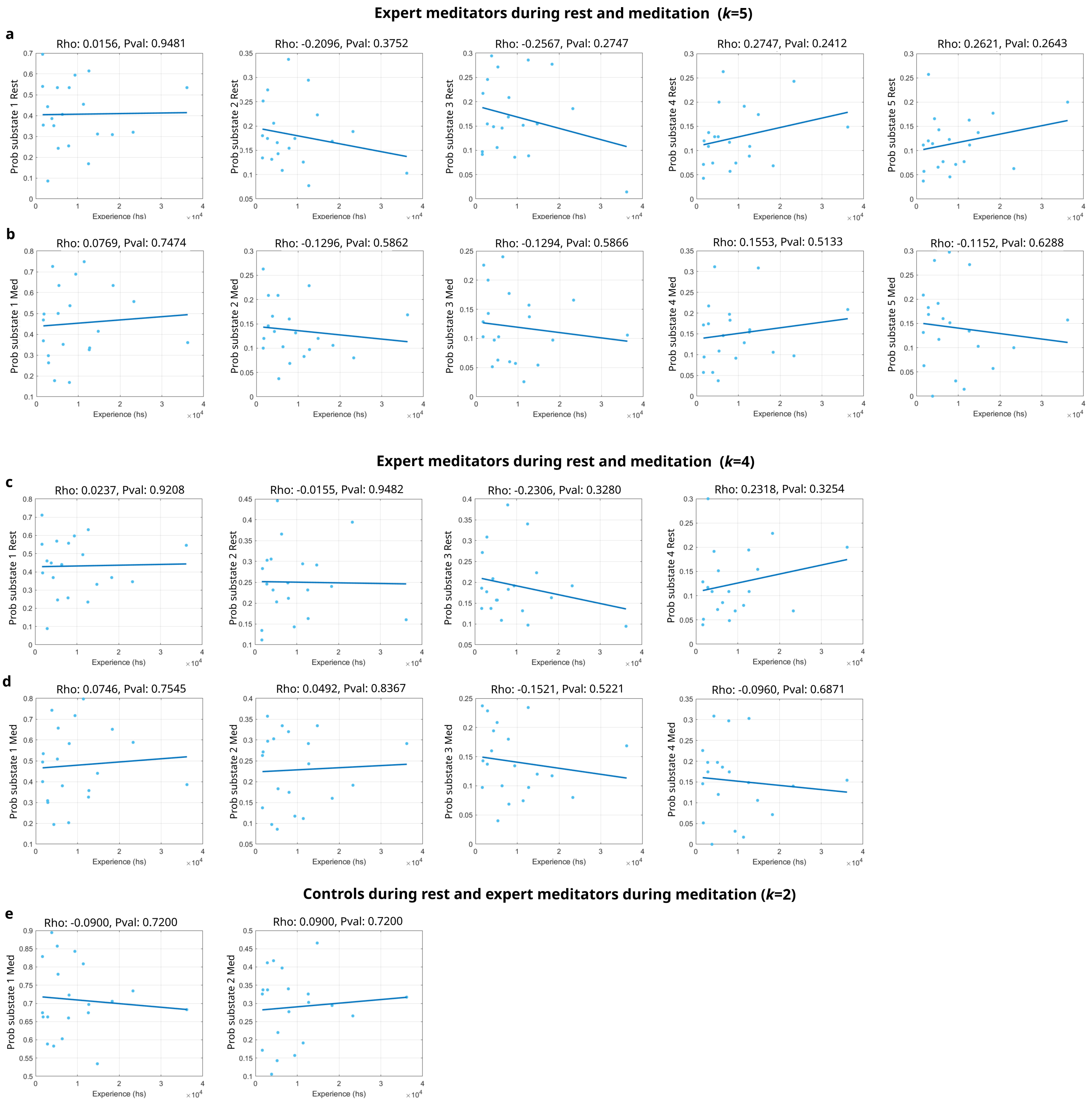

Figure. S 3. Correlation between the probability of occurrence of each substate from the PMS and experience of expert meditators. No significant correlation was found. **a** LEiDA on expert meditators during resting-state and meditation for k=5, PMS of rest. **b** LEiDA on expert meditators during resting-state and meditation for k=5, PMS of meditation. **c** LEiDA on expert meditators during resting-state and meditation for k=4, PMS of rest. **d** LEiDA on expert meditators during resting-state and meditation for k=4, PMS of meditation. **e** LEiDA on controls during resting-state and expert meditators during meditation for k=2, PMS of meditation.

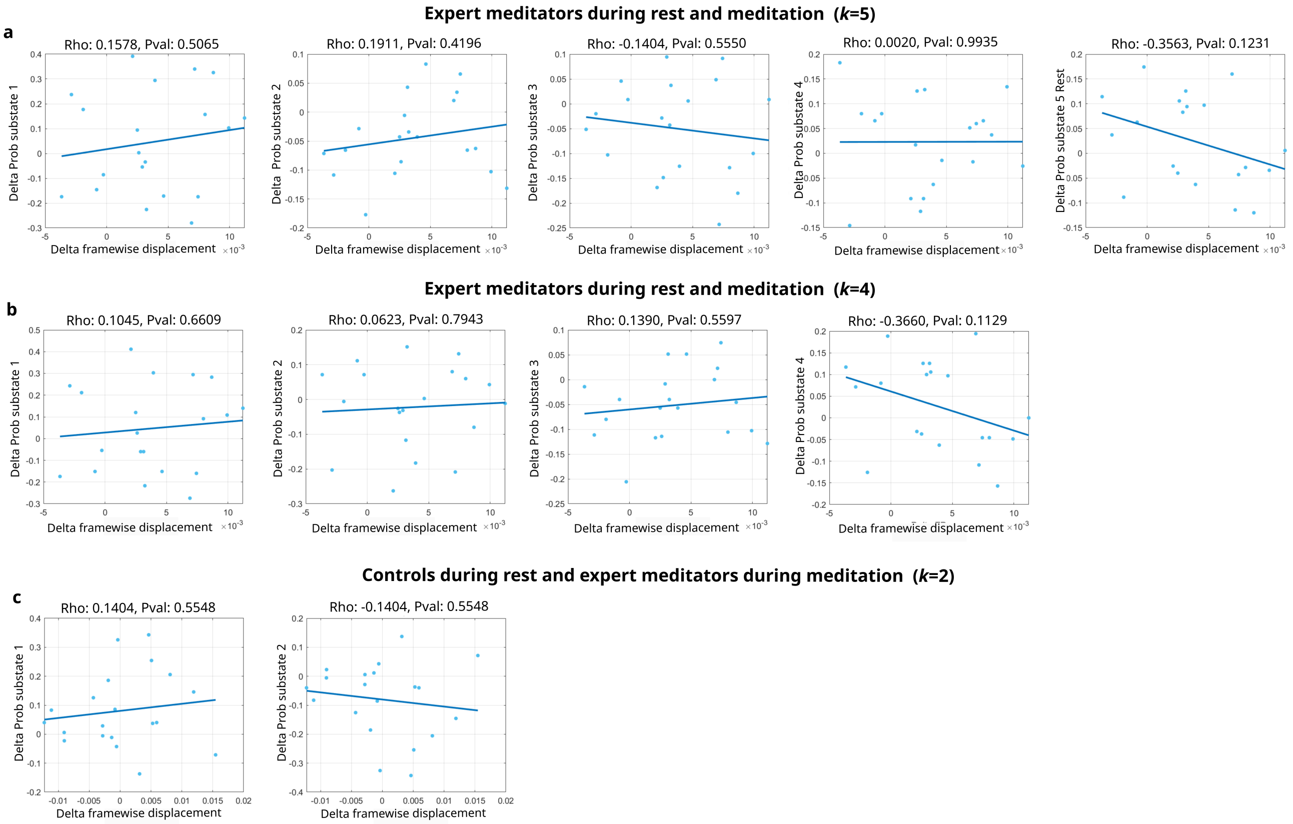

Figure. S 4. Correlation between scanning motion and substate occupancy. Correlation between the condition delta framewise displacement (meditation – rest) and the delta substate occupancy for each substate. No significant correlation was found, reflecting no relationship between the motion during the scan and the probability of any substate. **a** LEiDA on expert meditators during resting-state and meditation for *k*=5. **b** LEiDA on expert meditators during resting-state and meditation for *k*=4. **c** LEiDA on controls during resting-state and expert meditators during meditation for *k*=2.

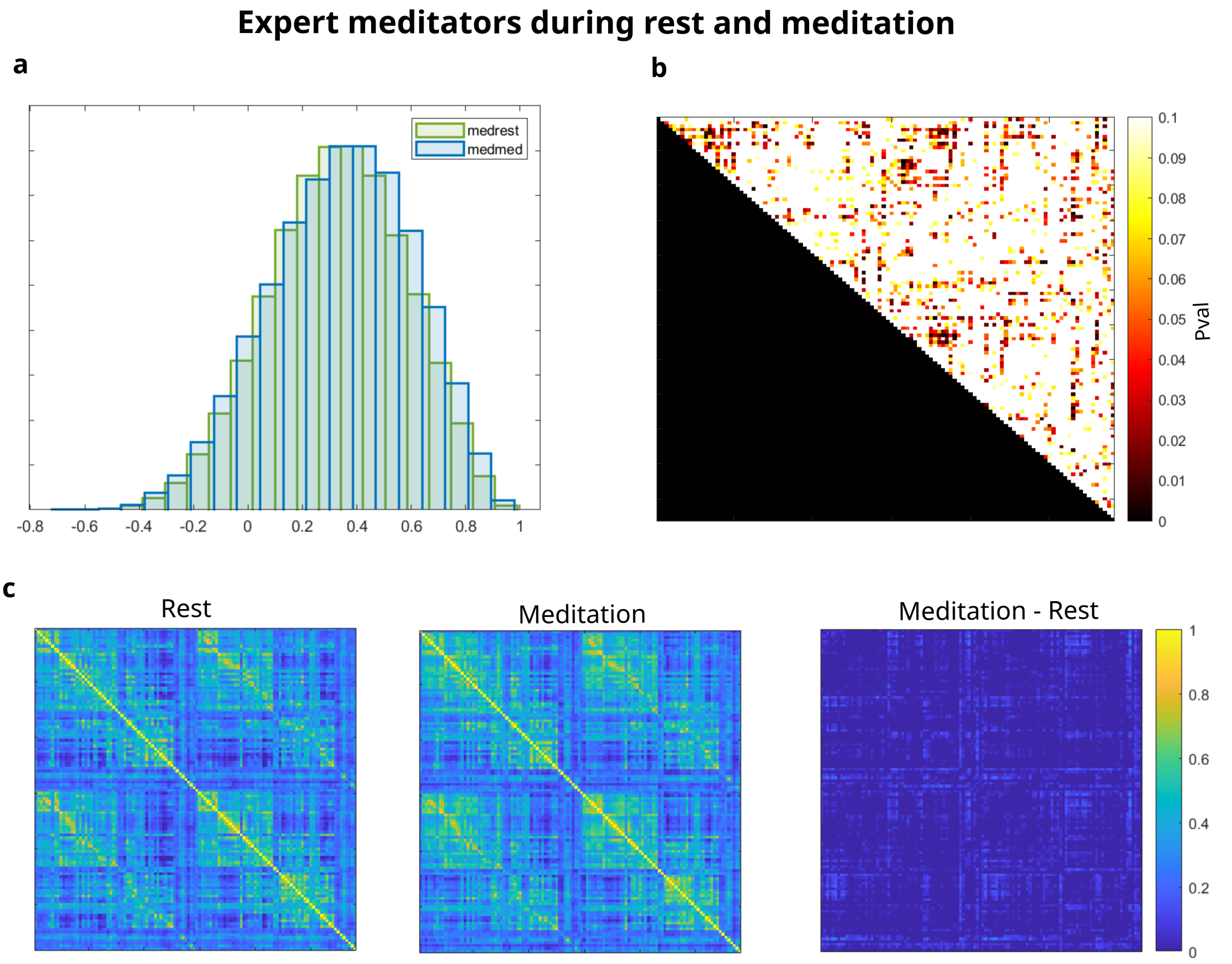

Figure. S 5. Static analysis of expert meditators during resting-state and meditation. Functional connectivity is not sensitive enough to characterize differences between the two brain states. **a** Histogram of the mean FC matrix of rest and mean FC matrix of meditation. The distributions closely overlap. **b** P-values of the node-to-node comparison of the FC matrices of all subjects in each brain state. None survive correction by multiple comparisons. **c** Visualization of mean FC matrices of rest, meditation, and their subtraction (rest-meditation). Very slight differences can be observed. The correlation between the mean FC of rest and the mean FC of meditation is significant and has a strength of 0.9106.

*

Controls during rest and expert meditators during meditation

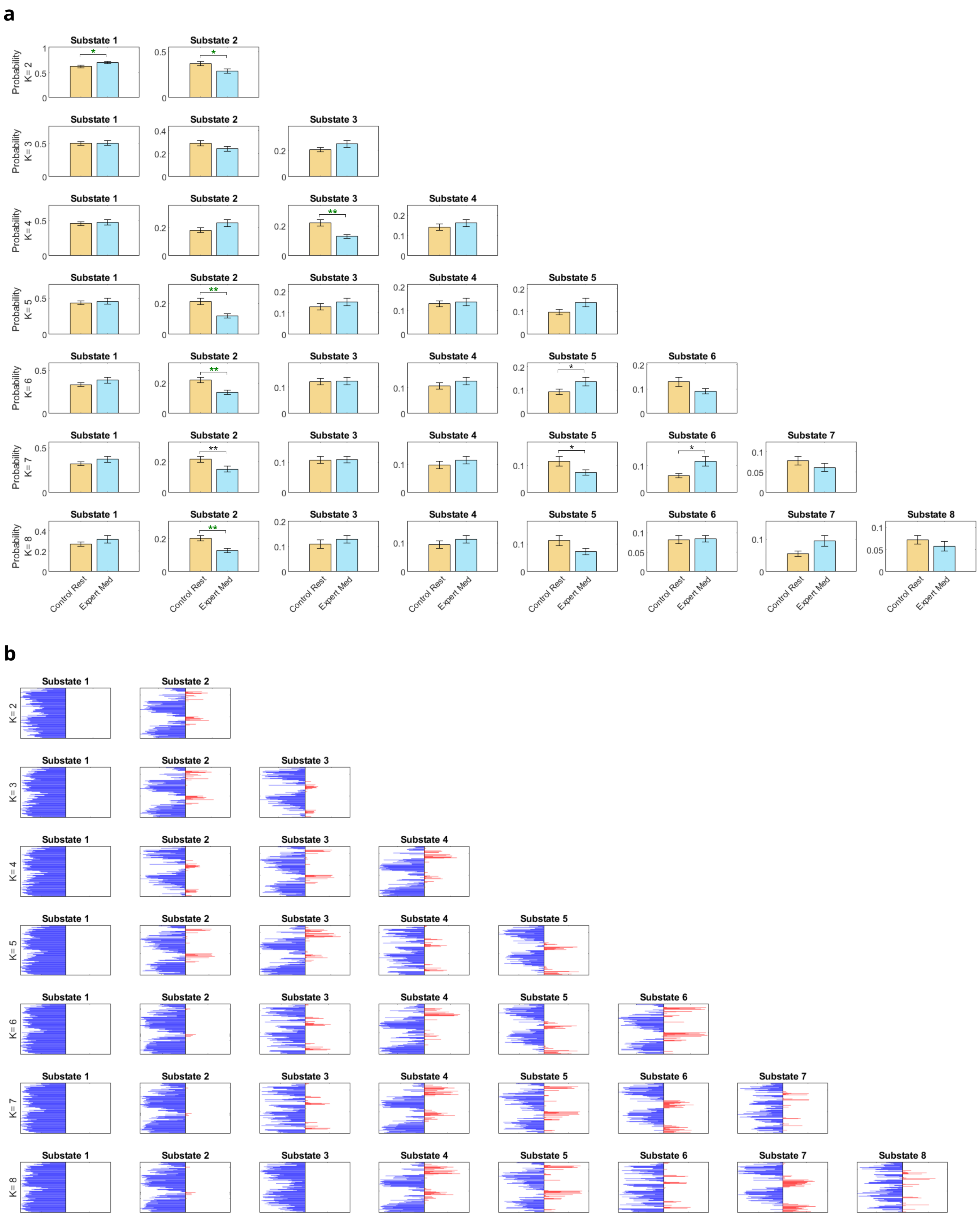

Figure. S 6.

Leading Eigenvector Dynamic Analysis (LEiDA) of controls during resting-state and expert meditators during meditation for all values of k. a Empirical Probabilistic Metastable Substate (PMS) Space of resting-state and meditation. Differences were computed with a 95% confidence interval and significance is represented with asterisks (* $p < 0.05$, ** $p < 0.01$ and *** $p < 0.001$). Differences surviving multiple comparison correction are represented in green, while those not surviving correction are represented in black. **b** Leading Eigenvectors for each substate of the PMS for all values of k.

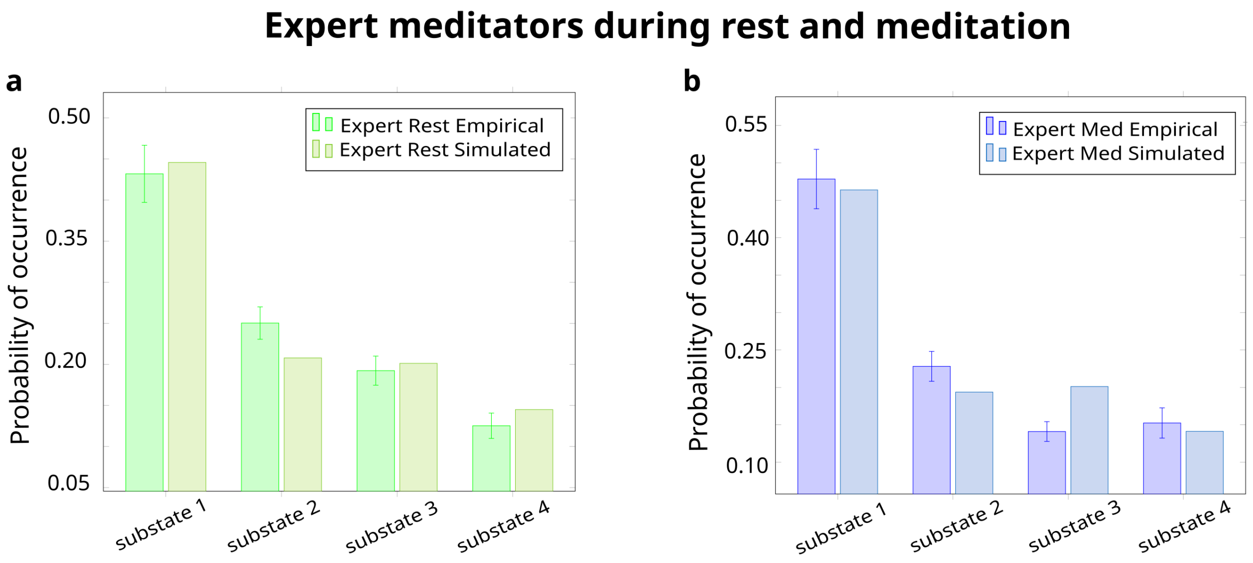

Figure. S 7. Model-based results for k=4: Whole-brain models in the analysis of expert meditators during resting-state and meditation. Empirical and simulated PMS for **a** resting state (G=0.04 with a KL distance of 0.0058) and **b** meditation (G=0.04 with a KL distance of 0.0141).

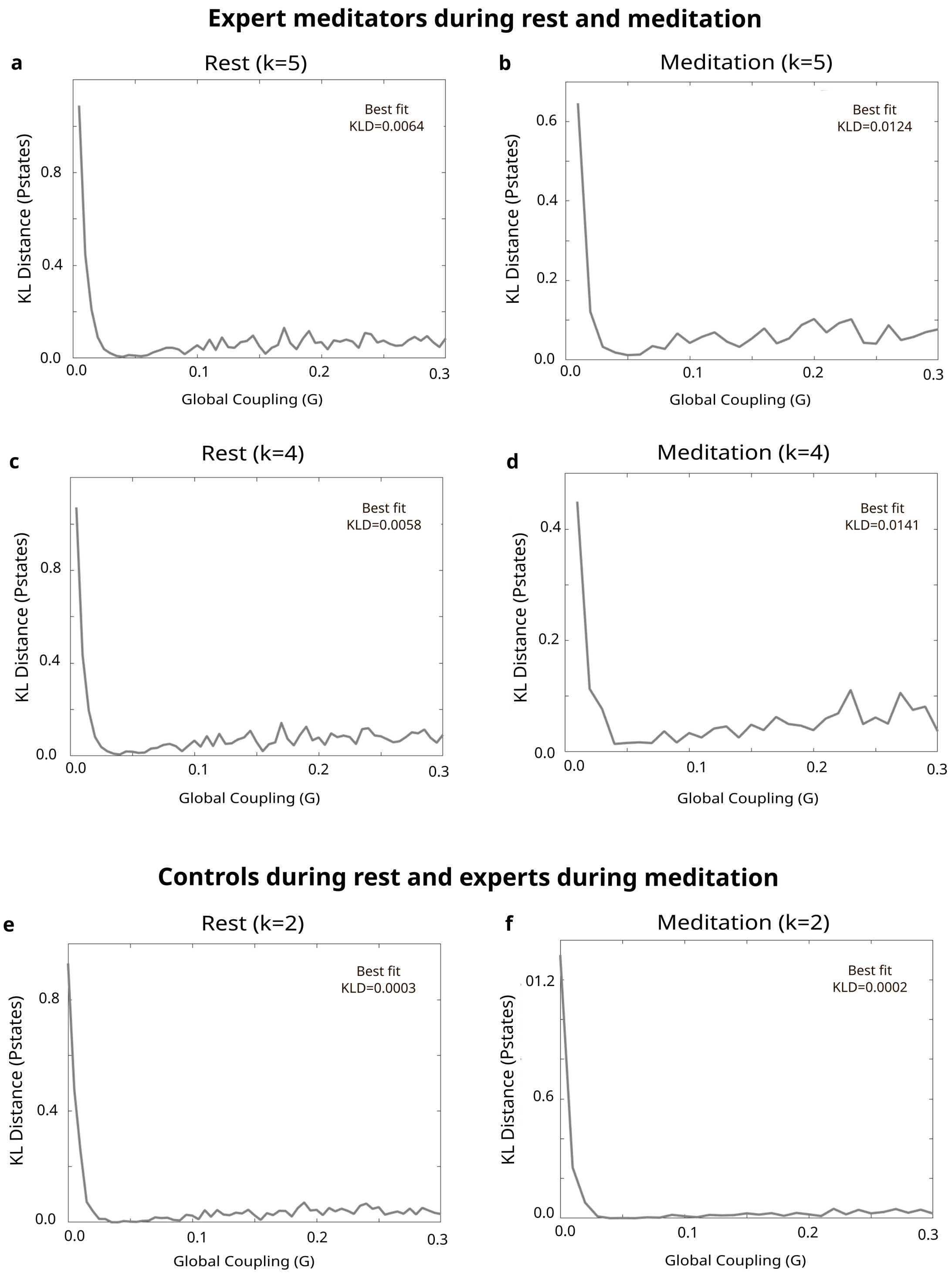
Expert meditators during rest and meditation
a
Rest (k=5)
Best fit
KLD=0.0064
b
Meditation (k=5)
Best fit
KLD=0.0124
c
Rest (k=4)
Best fit
KLD=0.0058
d
Meditation (k=4)
Best fit
KLD=0.0141
Controls during rest and experts during meditation
e
Rest (k=2)
Best fit
KLD=0.0003
f
Meditation (k=2)
Best fit
KLD=0.0002
KL Distance (Pstates)
Global Coupling (G)

Figure. S 8.

Model-based results: Fitting and optimization for each brain state. In the analysis of expert meditators during resting-state and meditation for k=5, **a** in rest G=0.04 (KLD=0.0064) and **b** in meditation G=0.05 (KLD=0.0122). In the analysis of expert meditators during resiting-state and meditation for k=4, **c** in rest G=0.04 (KLD=0.0058) and **d** in meditation G=0.04 (0.0141). In the analysis of controls during resiting-state and expert meditators during meditation for k=2, **e** in rest G=0.04 (KLD=0.0003) and **f** in meditation G=0.04 (0.0002).

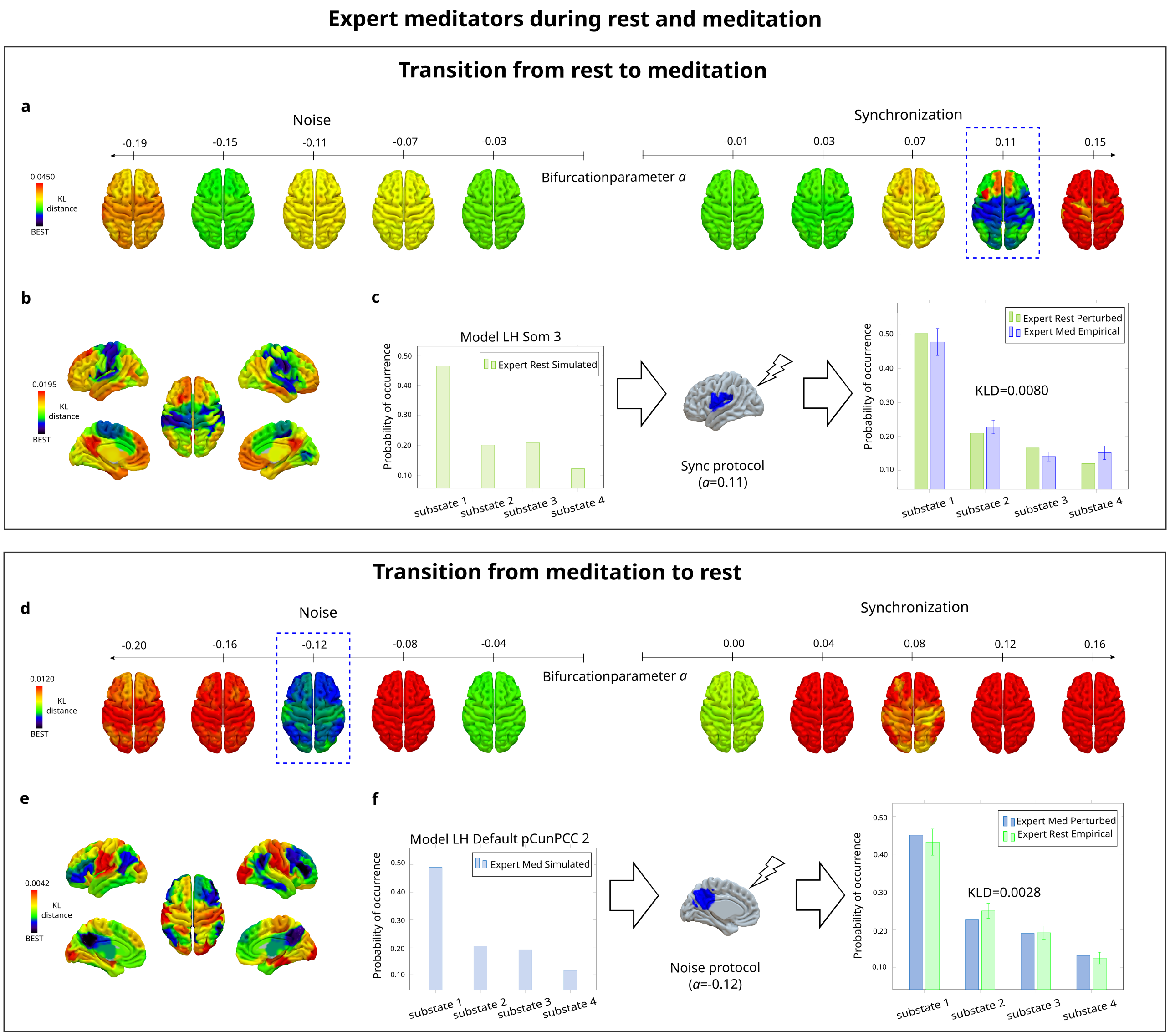

Figure. S 9. Model-based results for k=4: *In silico* stimulation in the analysis of expert meditators during resting-state and meditation. This shows the robustness of the perturbational approach for different values of k in regards to the identification of the most sensitive brain areas to promote a transition from resting-state to meditation in expert meditators and vice versa. **a** Transitions from rest towards meditation were possible for the synchronization protocol, characterized by lower KL distance between the perturbed modeled PMS of rest and the empirical PMS of meditation. **b** In the synchronization protocol, optimal perturbation for each brain area. The color bar represents the KL distances. **c** The best transition was found in the area LH Som 3 from Schaefer 100 parcellation (Schaefer et al., 2018) for a bifurcation parameter value of a=0.11. The perturbed rest PMS is closer to the target empirical meditation PMS. **d** Opposite transitions from meditation to rest were found in the noise protocol. **e** Optimal perturbation of each brain area in the noise protocol, at their particular simulation intensities, rendered onto brain maps. **f** Best transition was obtained at a bifurcation parameter value of a=-0.14 in the area LH Default PCunCC 2 from Schaeffer 100 parcellation (Schaefer et al., 2018). The PMS of the perturbed meditative state is closer to the PMS of the target empirical rest state.
